# Ultrasonic Assessment of Precursor Fatigue Damage in CFRP Composites: A Synthesis of Coda Waves, Nonlinear Indicators, Stress Relaxation, and Validation

**DOI:** 10.3390/s26196190

**Published:** 2026-09-29

**Authors:** Hossain Ahmed, Sourav Banerjee

**Affiliations:** 1 Department of Mechanical Engineering, Georgia Southern University, Statesboro, GA 30458, USA; 2 Department of Mechanical Engineering, University of South Carolina, Columbia, SC 29208, USA; banerjes@cec.sc.edu

**Keywords:** carbon fiber reinforced polymer, precursor damage, nonlinear ultrasonics, coda wave interferometry, guided waves, pulse-echo ultrasonics, stress relaxation, structural health monitoring

## Abstract

Carbon fiber-reinforced polymer composites can accumulate distributed microstructural damage before visible delamination or substantial stiffness loss becomes apparent. This article presents a program-based synthesis of six publications that examined precursor fatigue damage using guided coda waves, nonlinear guided waves, high-frequency pulse-echo ultrasonics, and scanning acoustic microscopy. The evidence comprises a separate incremental coda-wave campaign to 300,000 cycles and a coordinated fatigue-relaxation program conducted in three 75,000-cycle stages. Coda-wave interferometry detected progressive propagation changes while the first-arriving guided-wave packets remained largely unchanged. Nonlinear measurements showed changes in second- and third-order parameters and sideband peak counts, while the guided-wave nonlinear response decreased by as much as 26.8% during the 8 h unloaded period. This result demonstrates that post-loading measurement time must be controlled when transient stress-related and persistent damage-related responses are compared. Within the specific Route II material system and loading protocol, the 5 Hz specimens exhibited greater degradation than the 2 and 10 Hz specimens. This frequency ranking is not proposed as a general relationship for CFRP composites. Residual-strength testing, microscopy, and SAM provided convergent supporting evidence, but the overlapping datasets and small specimen groups do not constitute independent statistical replication. The proposed framework therefore demonstrates laboratory-scale sensitivity to precursor material-state changes but does not yet provide reliable prediction of residual strength, remaining fatigue life, or probability of failure. Field implementation will require larger independent datasets, uncertainty estimation, environmental compensation, repeated sensor installations, and validation on representative structural components.

## 1. Introduction

### 1.1. Early Stage Fatigue Damage in CFRP Composites

Carbon fiber reinforced polymer (CFRP) composites are widely used in aerospace, automotive, wind energy, marine, and civil structures because of their high specific strength, stiffness, and resistance to environmental degradation [1,2]. Their increasing use in primary load-carrying components requires reliable assessment of fatigue damage throughout structural service. Unlike metallic materials, where fatigue failure is often dominated by the initiation and growth of a principal crack, fatigue damage in CFRP composites develops through interactions among the matrix, fibers, interfaces, and ply boundaries [3,4].

Matrix microcracking, fiber and matrix debonding, local fiber breakage, ply splitting, void development, and immature interlaminar cracking may initiate independently and accumulate throughout the laminate [5,6]. During the initial fatigue stage, these defects alter local stress transfer, energy dissipation, and elastic properties while the laminate retains much of its global stiffness and load-carrying capacity. The measured structural response may therefore remain close to its pristine condition despite continued internal degradation. The duration and severity of this precursor stage depend on laminate architecture, constituent properties, manufacturing defects, stress ratio, loading frequency, and load history [7,8].

**Figure 1:**
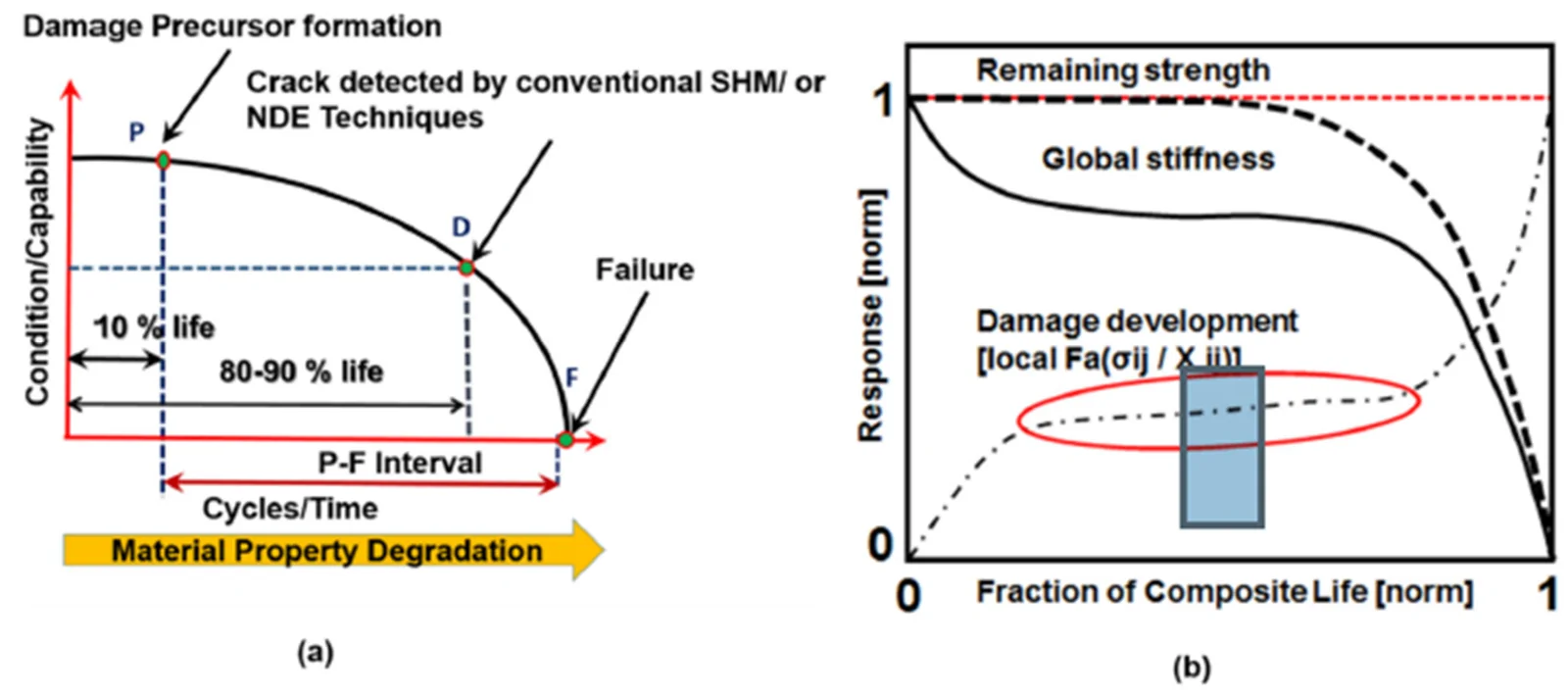
Conceptual representation of precursor fatigue damage in CFRP composites: (**a**) interval between the initiation of distributed damage at point (P) and its detection by conventional monitoring at point (D); and (**b**) development of local material damage while global stiffness and remaining strength exhibit limited initial changes. Adapted from [9].

Figure 1 illustrates the requirement for precursor damage assessment. Local damage begins near point (P), but conventional structural health monitoring or nondestructive evaluation may not provide a clear indication until point (D), when damage interaction and localization become more pronounced. The interval between these points provides an opportunity to identify material degradation before rapid damage growth and substantial loss of structural capability. The life fractions shown in the figure represent a conceptual damage progression rather than a universal fatigue law. Their values can change with material system, geometry, loading condition, and the criterion used to define damage initiation.

### 1.2. Challenges in Detecting Precursor Damage

In this synthesis, precursor damage is operationally defined as fatigue-induced material-state degradation occurring after the pristine baseline but before visible macroscopic delamination, unstable damage growth, or final structural failure. Within an individual experiment, its development is identified by a sustained change in an ultrasonic indicator across successive fatigue states, supported where available by microscopy, SAM, or residual-strength measurements. No universal numerical threshold or life fraction is assigned because baseline variability and damage sensitivity depend on the laminate, geometry, loading history, sensing configuration, and selected indicator. In the incremental CWI study, measurable changes became apparent at approximately 120,000 to 160,000 cycles, whereas visible delamination in the separate fatigue-life specimen began near 1 × 10^6^ cycles. The coordinated fatigue-relaxation studies examined material states through 225,000 cycles that retained macroscopic integrity but exhibited ultrasonic, microscopic, and mechanical evidence of degradation. Accordingly, the life fractions shown in Figure 1 are conceptual and should not be interpreted as universal boundaries between pristine, precursor-damage, and macroscopic-damage states.

Conventional nondestructive evaluation methods are effective for identifying delamination, impact damage, voids, and cracks when these defects produce measurable changes in geometry, acoustic impedance, or reflected wave energy. Their sensitivity decreases when fatigue degradation remains distributed as small matrix cracks, localized interfacial separation, or isolated fiber damage. The heterogeneous architecture of CFRP further complicates this assessment because fiber orientation, resin-rich regions, ply interfaces, porosity, fiber waviness, and local thickness influence ultrasonic propagation before fatigue damage is introduced [10,11].

Linear ultrasonic measurements generally assess changes in wave velocity, attenuation, amplitude, arrival time, or reflected energy. These quantities respond primarily to changes in effective elastic properties or the formation of distinct scattering boundaries. During the precursor stage, however, changes in global stiffness can remain close to experimental variability while microstructural damage continues to accumulate. Consequently, the first arriving guided wave packets may exhibit only minor changes until the defects become sufficiently large or concentrated to perturb the dominant propagation modes [12]. This limitation is particularly important for guided wave structural health monitoring, where modal behavior, propagation distance, geometry, and structural boundaries also affect the measured response [13,14,15].

Reliable assessment must also separate fatigue-induced material changes from environmental and measurement-related variability. Temperature, applied stress, moisture, boundary conditions, transducer response, and coupling can modify wave velocity, phase, amplitude, and modal content without persistent damage-related change. Bonded piezoelectric sensors introduce additional uncertainty because fatigue loading may affect both the composite and the sensor adhesive layer. Temperature effects can also depend on sensor placement, laminate thickness, excitation frequency, and wave mode [16]. A damage indicator may therefore provide high sensitivity without sufficient confidence regarding the physical origin of the measured change.

### 1.3. Development of Ultrasonic Methods for Precursor Damage Assessment

The limited sensitivity of direct linear wave features has motivated methods that extract information from scattered wavefields and nonlinear wave interactions. Coda wave interferometry (CWI) analyzes the later portion of the ultrasonic response formed through scattering, reflection, and mode conversion within a heterogeneous medium [12,17]. Small velocity perturbations accumulate along multiple propagation paths and appear as measurable phase changes within the coda [18,19]. CWI has therefore been used to monitor weak changes that remain difficult to resolve from the direct wave arrival, although its response remains sensitive to temperature, stress, and other environmental conditions [20].

Nonlinear ultrasonics provides a complementary measure of the material state. Microcracks, interfacial sliding, local debonding, and crack face contact distort the propagating waveform and generate higher harmonics or spectral modulation. These nonlinear components may change before measurable reductions in linear elastic properties occur [21,22,23]. Second harmonic generation has consequently been investigated as a material state indicator, although accurate measurements require control of source nonlinearity, diffraction, attenuation, coupling, and receiver response [24].

The propagation configuration determines the spatial scale of this nonlinear assessment. Guided Lamb waves interrogate an extended region between bonded sensors and are therefore suitable for structural monitoring [15]. High-frequency pressure waves provide shorter wavelengths and localized through-thickness interrogation but experience greater attenuation and require scanning access. The two configurations address different requirements: guided waves provide structural coverage, whereas high-frequency pulse-echo measurements provide localized information without relying on bonded sensors.

The ultrasonic response also depends on the mechanical state at the time of measurement. Following fatigue loading, viscoelastic deformation and internal stress redistribution produce partial recovery during an unloaded period. Measurements taken immediately after unloading may contain contributions from accumulated degradation and transient internal stress. Repeated measurements during relaxation are therefore required to determine which part of the ultrasonic response decreases with time and which part remains after the selected relaxation period. Mechanical testing, microscopy, and scanning acoustic microscopy are subsequently needed to determine whether these ultrasonic changes correspond to physical degradation and reduced structural capacity.

### 1.4. Scope and Synthesis Approach

This article presents a focused synthesis of six peer-reviewed publications from a coordinated investigation of precursor fatigue damage in woven CFRP composites. The studies used related material systems and fatigue protocols to examine incremental CWI, nonlinear guided waves, high-frequency pulse-echo ultrasonics, sideband analysis, stress relaxation, residual tensile strength, and microscopic damage. Some publications analyzed complementary measurements obtained from related experimental campaigns. Their results are therefore interpreted as mutually supporting evidence rather than statistically independent replications.

The objective is to determine how these methods contribute to precursor damage assessment across spatial, spectral, temporal, and mechanical scales. The synthesis first establishes the common experimental framework and then examines the physical basis, signal processing, experimental observations, and implications of each ultrasonic approach. The subsequent sections compare their sensing roles, evaluate mechanical and microscopic validation, and propose a staged strategy in which guided waves provide structural-scale monitoring and high-frequency measurements provide targeted confirmation. External literature is used to establish the scientific context and current research requirements; the article is not intended as a systematic review of all ultrasonic methods for CFRP composites.

### 1.5. Review Methodology and Evidence Boundaries

This article uses a program-based narrative synthesis to develop an integrated ultrasonic assessment framework. It is not a systematic review of the broader CFRP structural-health-monitoring literature. The experimental evidence set consists of six peer-reviewed publications produced between 2017 and 2025 through the coordinated research program examined here. These publications report experimental evidence concerning ultrasonic material-state assessment, fatigue-induced nonlinear response, post-fatigue relaxation, spatial material characterization, and mechanical and microstructural validation.

The six publications were not selected through a field-wide database search; therefore, a PRISMA screening process was not applied. External publications were used to establish the scientific context, compare the findings with developments in the broader field, and identify remaining research needs. They were not treated as part of the six-study experimental dataset. This program-based evidence boundary permits relationships among shared specimens, sensing methods, measurement times, and validation results to be examined without claiming systematic or comprehensive coverage of CFRP structural-health-monitoring research.

## 2. Development of an Integrated Ultrasonic Framework for Precursor Damage Assessment

The six studies synthesized in this article employed complementary ultrasonic mechanisms to characterize different manifestations of precursor fatigue damage in CFRP composites. Coda wave interferometry uses multiply scattered ultrasonic waves to detect small changes in effective elastic properties through relative velocity variation and waveform decorrelation [18,19]. Nonlinear ultrasonic methods measure harmonic generation, spectral changes, and nonlinear elastic interactions associated with fatigue-induced microstructural discontinuities [21,23]. High-frequency pulse-echo ultrasonics and scanning acoustic microscopy provide localized through-thickness characterization through nonlinear parameters, sideband peaks, acoustic velocity distributions, and damage entropy. These methods extend ultrasonic assessment beyond the detection of established defects by examining changes in the material state that occur before visible delamination or a substantial reduction in global stiffness.

The techniques differ in sensing scale, propagation mechanism, and measured response. Bonded PZT guided waves interrogate an extended region between the actuator and receiver. Coda wave interferometry analyzes the multiply scattered portion of the guided wave response to quantify distributed material state changes, whereas nonlinear guided wave analysis examines harmonic components generated within the directly propagating wavefield. High-frequency pulse-echo measurements interrogate localized regions through the laminate thickness without relying on bonded sensors. Scanning acoustic microscopy provides spatial maps of acoustic velocity and material heterogeneity. Post-fatigue relaxation measurements further distinguish transient stress-dependent changes from persistent degradation, while residual strength testing and microscopy provide supporting mechanical and microstructural evidence.

The studies were conducted through two experimental routes. Route I was a separate experimental campaign in which guided coda-wave measurements were collected during fatigue loading to 300,000 cycles. Route II was developed from a coordinated specimen program involving 22 woven CFRP specimens prepared according to ASTM D3039 [25]. Nine specimens were allocated to bonded-PZT pitch-catch measurements, nine were allocated to high-frequency pulse-echo/SAM measurements, three were used for pristine tensile characterization, and one was used to estimate fatigue life and identify the onset of visible delamination. The five Route II publications reported different measurements or analyses from this coordinated program and therefore do not represent five independent specimen populations. The 5 and 15 Hz entropy investigation used specimens from the same CFRP source but is treated as a separate measurement campaign and is not quantitatively pooled with the 2, 5, and 10 Hz comparisons. Table 1 summarizes the specimen allocation and relationships among the source publications.

**Figure 2:**
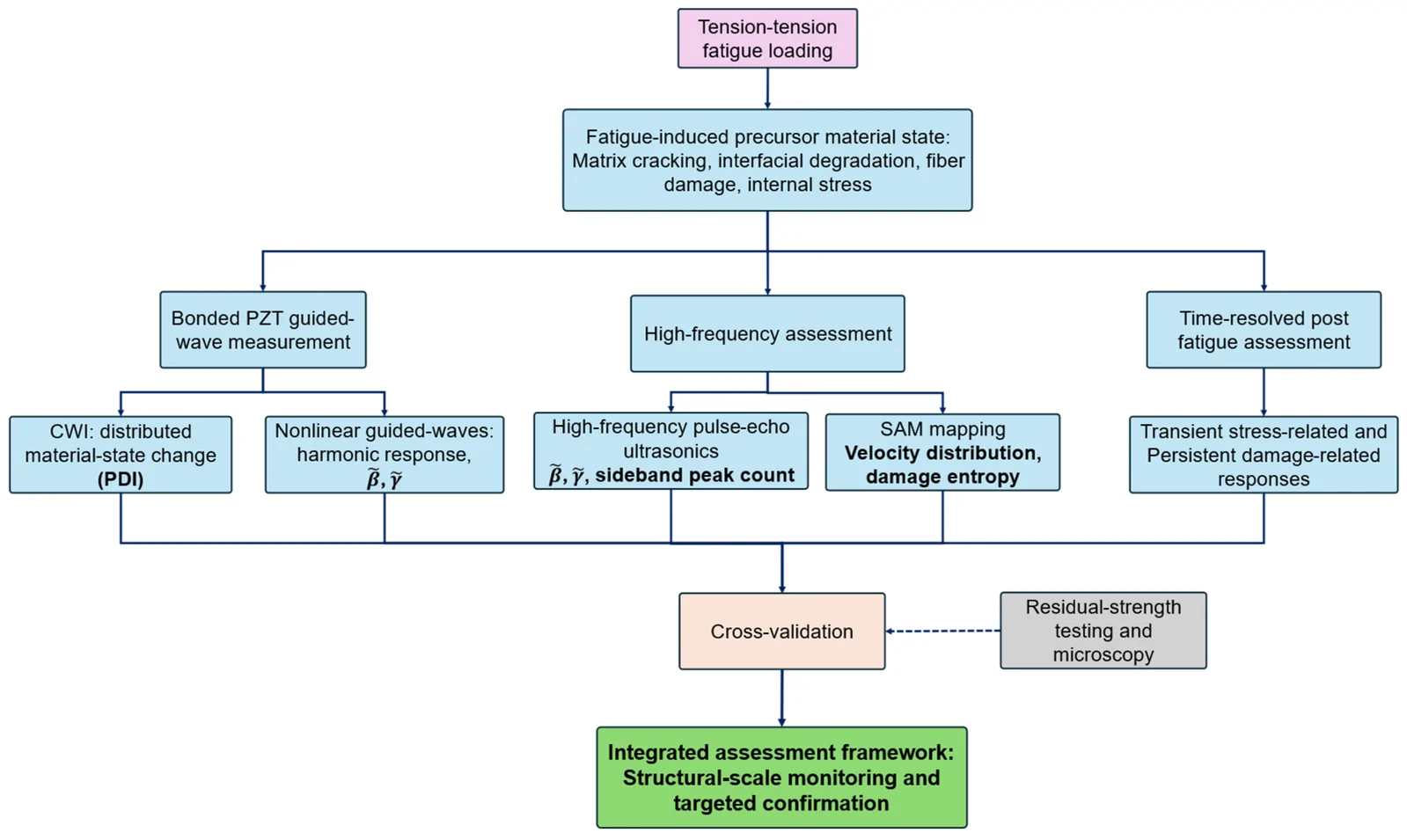
Conceptual framework linking fatigue-induced precursor material states with the ultrasonic sensing mechanisms and validation methods synthesized in this article.

Figure 2 presents the framework used to organize this synthesis. It connects the fatigue-induced precursor material state with the sensing scale and physical response measured by each ultrasonic method. Structural-scale guided-wave measurements provide distributed monitoring, high-frequency measurements provide localized confirmation, and time-resolved measurements clarify the influence of post-fatigue relaxation. Mechanical testing and microscopy support cross-validation of ultrasonic observations. This organization provides the basis for the subsequent assessment of each technique and for the integrated monitoring strategy proposed later in the article. Table 1 identifies the six source studies, their fatigue routes, and their specific contributions to this synthesis.

## 3. Experimental Framework

The six studies examined four-layer woven carbon-fiber-reinforced polymer specimens prepared from the same material system and with the same nominal geometry. However, they did not constitute six independent experimental datasets. Route I was a separate incremental CWI campaign conducted to 300,000 cycles. The remaining five publications drew on a coordinated Route II specimen program involving pitch-catch guided-wave specimens, pulse-echo/SAM specimens, pristine tensile specimens, and a fatigue-life specimen. Route II used three 75,000-cycle fatigue stages, each followed by an 8 h unloaded measurement period. Table 1 distinguishes the experimental units, measurement subsets, and dataset relationships reported by the individual publications.

### 3.1. Preparation of Cross-Ply CFRP Specimens

The specimens were prepared from commercially available four-layer carbon-fiber-reinforced woven composite plates manufactured using carbon-fabric substrates and an epoxy resin system. Each fabric layer contained 3k carbon-fiber tows woven in the [0,90^°^] directions. The thickness of each lamina was reported as ∼280 μm, and the plate density reported by the supplier was 1605 kg/m^3^. The specimens were machined according to ASTM D3039 with final dimensions of 250 mm in length, 25 mm in width, and 1.5 mm in thickness. The incremental CWI investigation was conducted as a separate experimental study, and its specimen count is therefore reported independently. For the coordinated Route II program, 22 specimens were prepared according to ASTM D3039. Nine specimens were reserved for bonded-PZT pitch-catch experiments, with three assigned to each of the 2, 5, and 10 Hz loading conditions. Nine additional specimens were reserved for pulse-echo/SAM experiments, again with three assigned to each loading condition. Of the remaining four specimens, three were tested monotonically to determine the pristine tensile response, and one was subjected to extended fatigue loading to estimate fatigue life and identify the onset of visible delamination.

The individual Route II publications reported different portions of this specimen allocation. Patra et al. reported 21 specimens in the strength-recovery study because the fatigue-life specimen was not included in that paper. Ahmed et al. reported 13 specimens in the high-frequency study because that publication focused on the nine pulse-echo/SAM specimens, the three pristine tensile specimens, and the fatigue-life specimen. These differences in reported totals reflect publication scope rather than separate specimen populations.

Glass-fiber-reinforced polymer tabs were bonded to the ends of the CFRP specimens to transfer the tensile load into the gauge section and reduce damage near the grips. Epoxy 9340 was used to attach the tabs, and the adhesive was allowed to cure for 48 h. Before fatigue loading, separate specimens were loaded monotonically in tension to establish the stress–strain response and ultimate tensile strength of the material. In the coordinated fatigue-relaxation studies, the average ultimate tensile load of the three pristine tensile specimens was approximately 8400 lbf. The maximum fatigue load was set at 60% of this value.

For guided-wave measurements, two piezoelectric wafer active sensors were bonded to the surface of each specimen using Epoxy 9340. The sensors were placed on opposite sides of the specimen centerline in a pitch–catch arrangement with a center-to-center separation of 90 mm. The adhesive thickness was measured as ∼150 μm, and the bonded sensors were allowed to cure at room temperature for three days before testing. Specimens assigned to scanning acoustic microscopy did not contain bonded PZT sensors. This separation allowed the PZT-based guided-wave measurements to be compared with pressure-wave measurements that were not affected by the sensor bond layer.

### 3.2. Mechanical Fatigue Loading Protocol

Tension–tension fatigue loading was applied using an MTS servo-hydraulic testing machine. The loading ratio was defined as

$$=\frac{\sigma_{\min}}{\sigma_{\max}}=0.01.$$

The maximum load used in the fatigue–relaxation tests was 60% of the average ultimate tensile load. The measurements were limited to the early portion of fatigue life, before the formation of large delamination or macroscopic fracture. Two fatigue-loading routes were used to examine the material response at different cycle intervals.

Route I: Incremental CWI Testing to 300,000 Cycles

Route I was used to examine the accumulation of precursor damage using guided coda waves. Five CFRP specimens were monitored during tension-tension fatigue loading at 10 Hz. Fatigue loading was interrupted after each block of 5000 cycles and guided-wave signals were recorded using the bonded PZT actuator-receiver pair. This loading and acquisition sequence continued to 300,000 cycles, producing 61 material states for each monitored specimen, including the pristine state.

**Figure 3:**
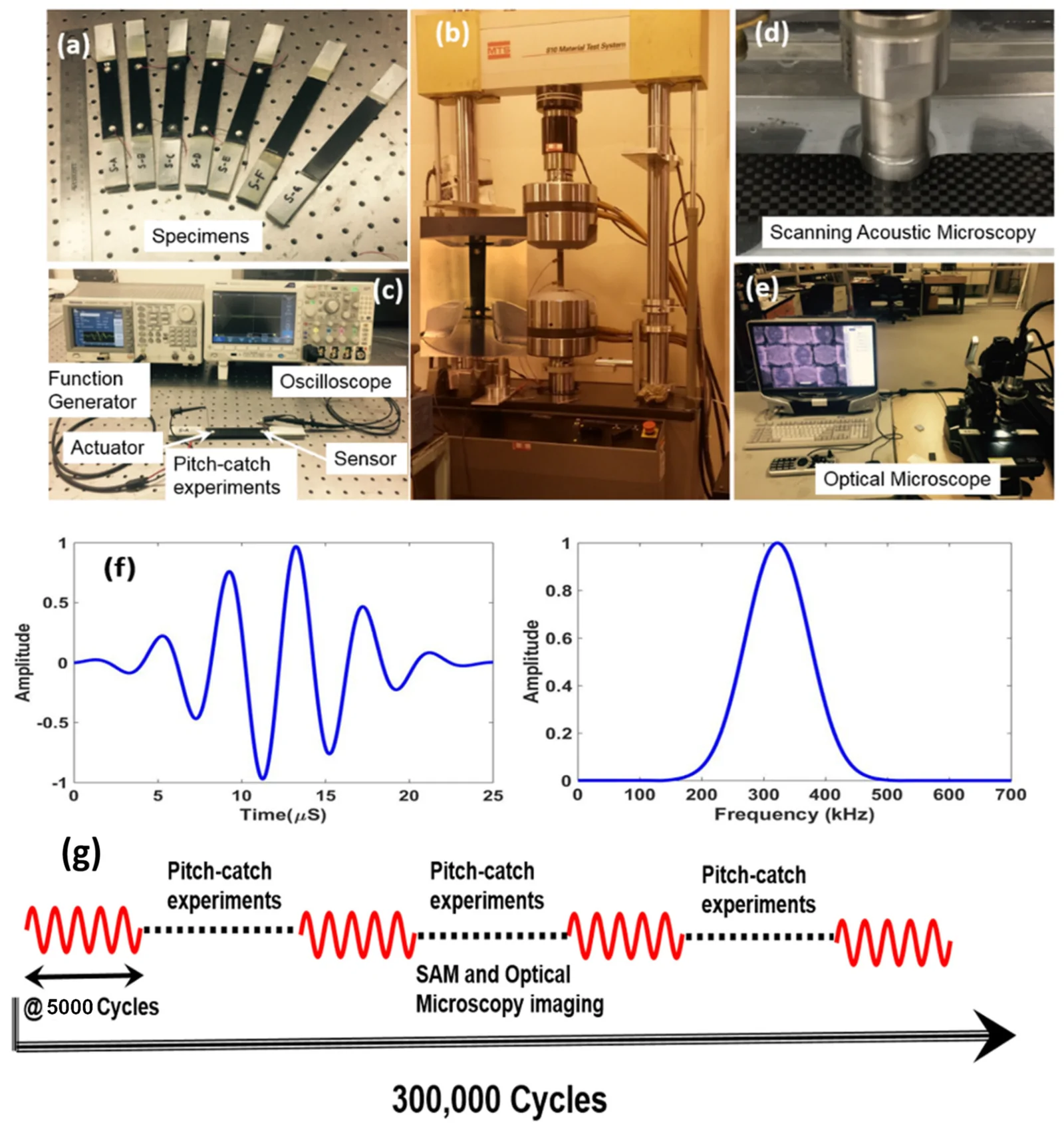
Experimental configuration and measurement sequence used for Route I: (**a**) woven CFRP specimens; Specimens S-A to S-E were used for pitch-catch experiments and S-F to S-G were used for scanning acoustic microscopy (**b**) MTS fatigue-testing system; (**c**) bonded PZT pitch-and-catch configuration; (**d**) scanning acoustic microscopy; (**e**) optical microscopy; (**f**) five-cycle tone burst excitation with a center frequency of 324 kHz and its corresponding frequency spectrum; and (**g**) incremental guided-wave acquisition at 5000-cycle intervals up to 300,000 cycles. Adapted from [9].

A separate fatigue-life specimen was used to determine the loading range for precursor-damage assessment. Visible delamination began near 1 × 10^6^ cycles, and the specimen remained intact until 2 × 10^6^ cycles. The 300,000-cycle limit was therefore selected to examine changes that occurred before visible delamination and final failure. The measurements obtained at each 5000-cycle interruption represented successive material states used in the CWI analysis. One additional specimen fatigued to 300,000 cycles was subsequently tested to tensile failure for residual-strength validation. Because this validation result was obtained from a single fatigued specimen, it is treated as supporting mechanical evidence rather than a population-level strength estimate. Figure 3 summarizes the experimental configuration and incremental measurement sequence used for Route I.

Route II: Three-Stage Fatigue–Relaxation Testing to 225,000 Cycles

**Figure 4:**
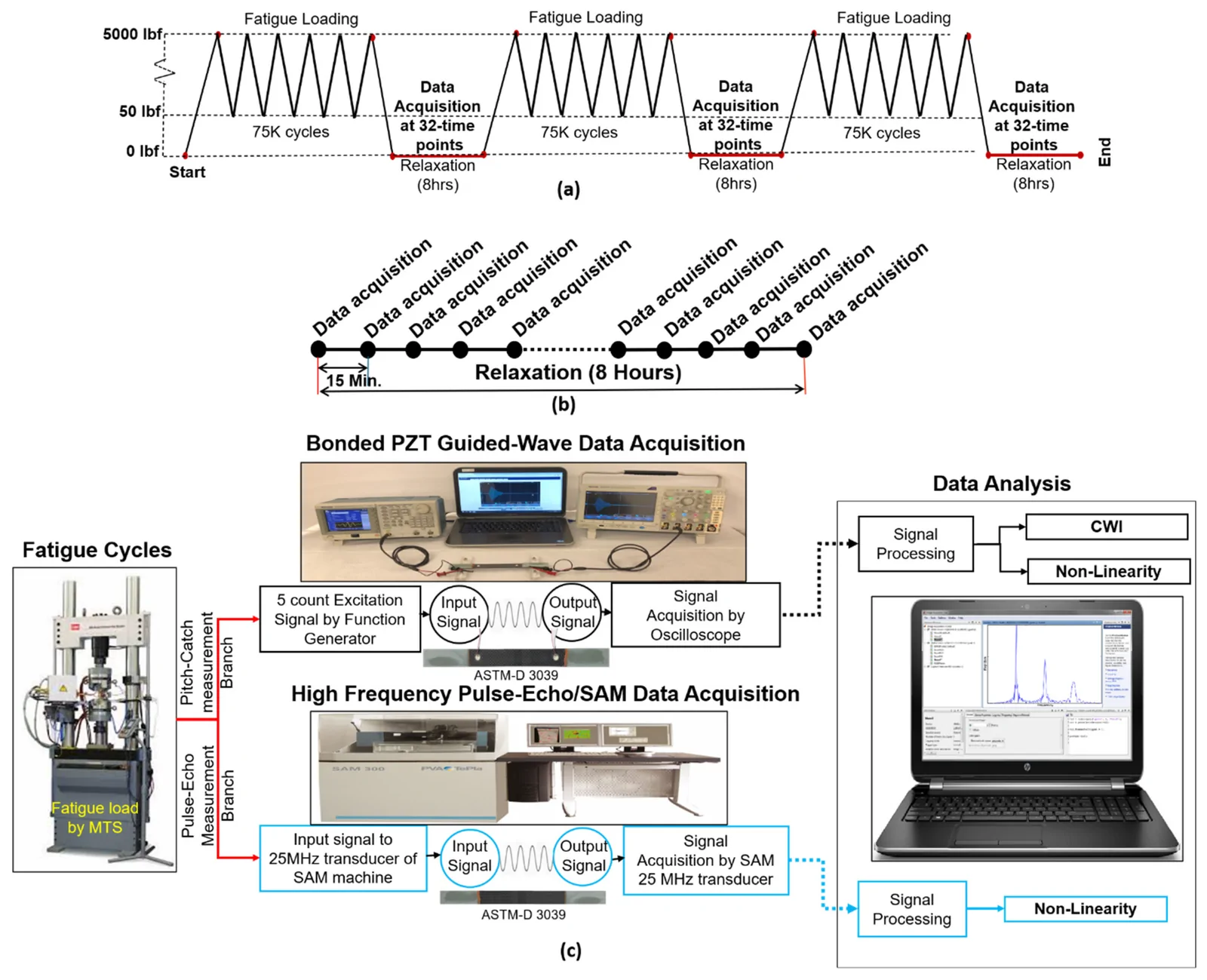
Three-stage fatigue–relaxation protocol and complementary ultrasonic measurement branches used in Route II: (**a**) three successive blocks of 75,000 tension–tension fatigue cycles, each followed by an 8 h unloaded relaxation period; (**b**) ultrasonic measurements acquired at 15 min intervals during each relaxation period; and (**c**) bonded-PZT guided-wave and high-frequency pulse-echo/SAM measurement branches used to evaluate time-dependent changes in the ultrasonic response. Adapted from [28].

Route II divided the fatigue history into three loading stages. Each specimen was first subjected to 75,000 tension–tension fatigue cycles and was then removed from the MTS machine for an 8 h unloaded period at room temperature. The specimen was returned to the testing machine for a second block of 75,000 cycles, producing a total of 150,000 cycles, followed by another 8 h unloaded period. The same procedure was repeated for the third block, producing a final total of 225,000 cycles. The complete three-stage loading, relaxation, and measurement sequence is summarized in Figure 4.

Within the coordinated Route II studies, nine specimens were assigned to pitch-catch guided-wave measurements and nine were assigned to high-frequency pulse-echo/SAM measurements. Each measurement route included three specimens at 2 Hz, three at 5 Hz, and three at 10 Hz. All specimens underwent the same three-stage loading and relaxation sequence. The entropy-based SAM study reported three specimens at 5 Hz and three specimens at 15 Hz. Although those specimens were prepared from the same CFRP material source, the 5 and 15 Hz study is treated as a separate measurement campaign. Its frequency-dependent results are therefore discussed qualitatively and are not combined statistically with the 2, 5, and 10 Hz results.

### 3.3. Relaxation and Measurement Sequence

Ultrasonic measurements in Route II were performed during the unloaded period following each 75,000-cycle fatigue block. Measurements were collected immediately after unloading and then every 15 min for 8 h. The procedure generated 33 measurement states during each relaxation period, including the first measurement at the beginning of relaxation. After the 8 h measurement period, the specimen was returned to the MTS system for the next fatigue block.

The measurement time after unloading was controlled because the ultrasonic response changed during stress relaxation. The polymer matrix and fiber–matrix interfaces continued to redistribute internal stress after removal of the mechanical load. Changes measured immediately after fatigue loading therefore contained contributions from both fatigue-induced material degradation and recoverable internal stress. Measurements obtained during the following 8 h were used to examine the time-dependent part of the response. The same measurement intervals were used after 75,000, 150,000, and 225,000 cycles, allowing changes caused by additional fatigue loading to be separated from changes occurring during the unloaded period.

### 3.4. PZT-Based Guided-Wave Acquisition

Guided Lamb waves were generated and received using two circular PZT sensors arranged in a pitch-and-catch configuration. The 7 mm diameter sensors were manufactured by STEMiNC, Steiner & Martins, Inc., (Davenport, FL, USA) and bonded to the CFRP specimens using Epoxy 9340. The fatigue–relaxation studies used PZT-5H sensors with an average capacitance of ~1.2 nF. One sensor acted as the actuator, while the second received the wave after propagation through a 90 mm path in the central region of the specimen.

For Route I, a function generator (Tektronix AFG3021C, Tektronix, Beaverton, OR, USA) generated a five-count tone burst at 324 kHz and 20 V peak-to-peak. The received signals were recorded using a Tektronix MDO3024 oscilloscope (Tektronix, Beaverton, OR, USA) at 50 MS/s, with 10,000 data points per waveform. Measurements were collected after each 5000-cycle fatigue block. For Route II, five-cycle tone bursts with center frequencies of 320 kHz and an excitation amplitude of 20 V peak-to-peak were used. A 512-point moving-average filter was applied to each recorded waveform to smooth rapid fluctuations and reduce high-frequency noise. Measurements were recorded every 15 min during the 8 h unloaded period following each 75,000-cycle fatigue stage. Sensor capacitance was monitored to identify possible changes in PZT performance.

The first arriving Lamb wave packets were used for conventional and nonlinear guided wave analyses, while the later coda portion was used for CWI. Because fatigue loading could also affect the bonded PZTs and adhesive layers, separate specimens without attached sensors were examined using high-frequency pulse-echo measurements.

### 3.5. Scanning Acoustic Microscopy and Measurements Without Bonded Sensors

Scanning acoustic microscopy was used to acquire ultrasonic data without attaching PZT sensors to the CFRP surface. The specimen was placed in distilled water, and a focused ultrasonic transducer was moved over a selected region of the gauge section. The transducer emitted a pressure-wave pulse and received the reflections from the specimen.

In the entropy-based study, an area of 2 × 1 in.^2^ was scanned using a grid of 125 × 70 points. One time-domain waveform was collected at each point, producing 8750 waveforms for each scan. Scans were completed every 15 min during each 8 h unloaded period after 75,000, 150,000, and 225,000 cycles. The procedure was applied to specimens loaded at 5 and 15 Hz.

The pressure wave first interacted with the water–composite interface and produced the front-surface reflection. The transmitted portion propagated through the laminate and reflected from the composite–water interface on the opposite surface. The time between the front- and back-surface reflections represented the through-thickness travel time. Using the specimen thickness, the local longitudinal-wave velocity was calculated at each point. The spatial distribution of velocity was then used to examine changes in the internal material state during fatigue loading and relaxation.

### 3.6. High-Frequency Ultrasonic Measurements

High-frequency measurements were performed in immersion pulse-echo mode using a 25 MHz transducer. For the high-frequency P-wave study, the transducer was positioned 35 mm above the specimen surface. The specimens were placed in room-temperature distilled water within 5 min after removal from the fatigue-testing machine. The central part of each specimen was scanned using 125 points along the longitudinal direction and 75 points across the width, producing 9375 measurement locations. Three specimens were examined at each fatigue frequency. At each point location, the recorded waveform contained 10,000 sampled data points. A 512-point moving-average filter was applied to smooth the waveform and reduce high-frequency noise.

The acquired waveform contained the front-surface reflection and the back-surface reflection after propagation through the 1.5 mm specimen thickness. The recorded signal length was 76 μs, while the time interval containing the front- and back-surface reflections was 1.5 μs. The time-domain and frequency-domain signals were used to extract travel time, wave velocity, fundamental and higher-harmonic amplitudes, normalized second- and third-order nonlinear parameters, and sideband peaks. These measurements were repeated every 15 min during the 8 h unloaded period after each fatigue stage.

### 3.7. Post-Fatigue Tensile Testing and Material Characterization

After completion of the ultrasonic measurements at 225,000 or 300,000 cycles, the fatigued specimens were loaded monotonically to tensile failure. Their residual tensile strengths were compared with the tensile strength of the pristine CFRP specimens. The fatigue-loaded specimens were also examined using optical microscopy, scanning electron microscopy, and scanning acoustic microscopy. These methods were used to observe matrix cracking, fiber–matrix debonding, voids, isolated fiber breakage, and edge delamination.

The ultrasonic results are presented in the following sections without using residual tensile strength as part of the initial interpretation. The tensile and microstructural results are examined later as supporting evidence for comparing the precursor-damage indicators obtained from CWI, nonlinear guided waves, high-frequency P-waves, and entropy-based measurements.

### 3.8. Replication and Interpretation of Measurement Variability

Experimental replication and repeated measurements served different purposes in source studies. The incremental CWI study monitored five specimens at 61 successive material states, including the pristine state. These measurements characterized progression within each specimen over the fatigue history. In the coordinated fatigue-relaxation program, each loading frequency was represented by three pitch-catch specimens and three pulse-echo/SAM specimens. The specimen groups therefore provided the basis for comparisons among the 2, 5, and 10 Hz conditions, while the repeated temporal and spatial measurements characterized changes within each specimen.

The acquisition and processing procedures were selected to reduce short-term measurement variability. Guided-wave measurements used a consistent excitation signal, fixed actuator-receiver spacing, monitored PZT capacitance, and the same smoothing procedure throughout the experiment. High-frequency measurements used a fixed water path and scan grid. Each recorded waveform contained 10,000 sampled data points, and a 512-point moving-average filter was applied to smooth rapid fluctuations and reduce high-frequency noise. The pristine control exhibited less than approximately 0.1% variation during the 8 h observation period, indicating short-term stability under the controlled laboratory conditions.

The source publications did not report specimen-level results in a common form that would permit consistent retrospective calculation of standard deviations, confidence intervals, or coefficients of variation across all ultrasonic indicators. The present synthesis therefore reports the specimen counts, available measures of variation, and experimental controls documented in the source studies. Comparisons among the 2, 5, and 10 Hz conditions are interpreted as trends within the tested specimen groups rather than as population-level statistically significant differences. Establishing transferable uncertainty bounds and detection thresholds will require larger specimen populations and dedicated evaluation of specimen, sensor, installation, and measurement-session variability.

## 4. Coda-Wave Interferometry for Precursor Damage Assessment

### 4.1. Physical Basis of Guided Coda-Wave Interferometry

**Figure 5:**
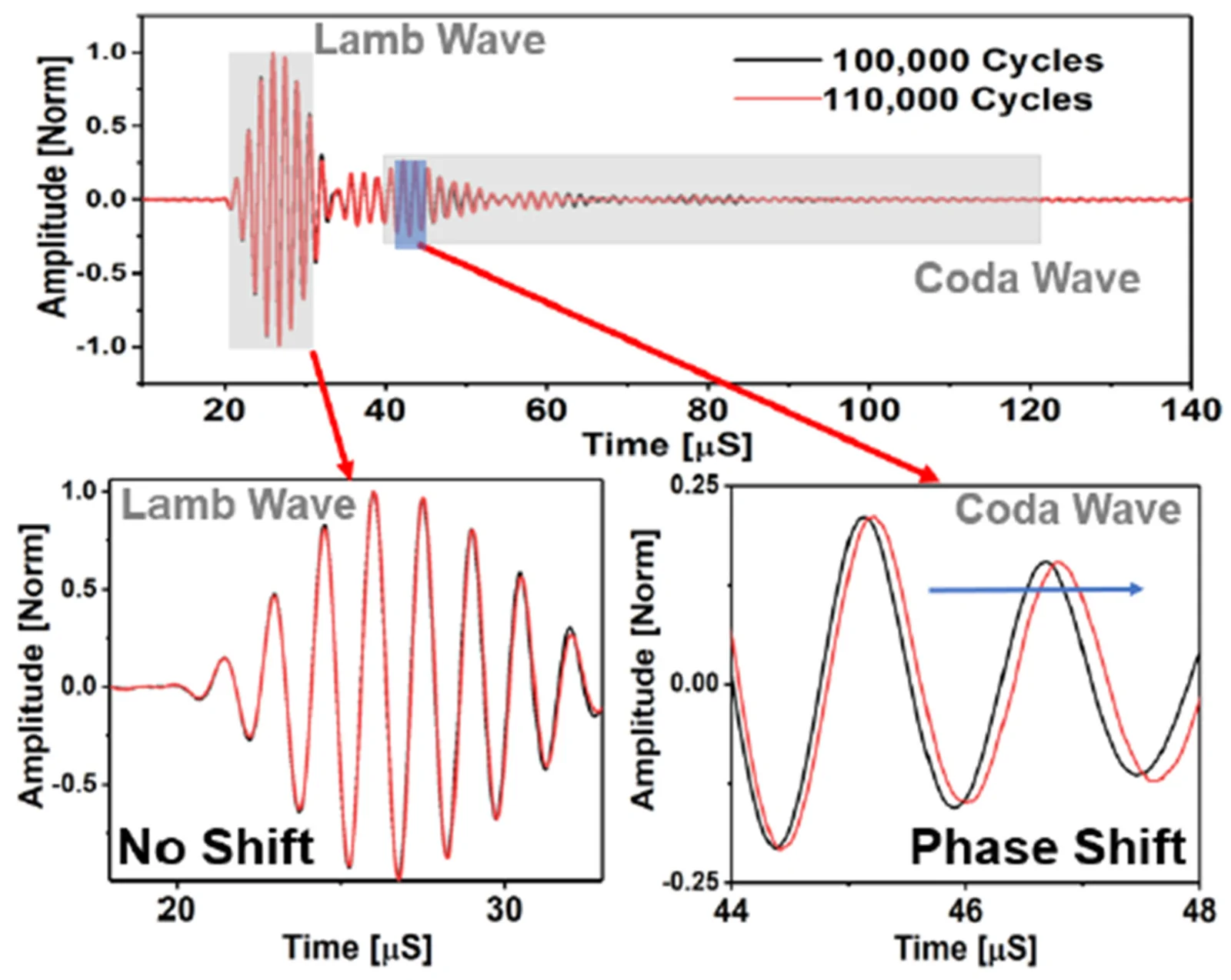
A typical comparison between two sensor signals obtained after two consecutive material states, which shows that the first arrival of Lamb wave signals is unaffected, but the coda wave signals are time-shifted. Adapted from [9].

Conventional guided-wave inspection primarily interprets changes in the first-arriving Lamb-wave packets through variations in arrival time, amplitude, or wave velocity. However, the first-arriving Lamb wave packets exhibited limited sensitivity during the investigated precursor regime. As illustrated in Figure 5, the first-arriving wave packets remained nearly unchanged between consecutive material states, whereas measurable temporal shifts developed in the coda portion of the signals. Guided CWI therefore analyzed the later coda, in which scattering, reflection, and mode conversion produce phase changes that accumulate over multiple propagation paths. This cumulative response allows fatigue-induced changes in the effective material properties to be resolved even when corresponding changes in the direct wave packets remain small [18,19,31].

The present investigation employed an incremental implementation of guided CWI to monitor precursor fatigue damage in woven CFRP laminates. Guided-wave signals acquired after every 5000 fatigue cycles were compared with the immediately preceding measurement rather than the pristine reference state. This strategy preserved waveform correlation throughout fatigue loading while enabling continuous quantification of material-state progression.

### 4.2. Signal Processing and Quantification of Material-State Changes

Successive material states were quantified using the stretching formulation of coda-wave interferometry [32]. Guided-wave signals were acquired after every 5000 fatigue cycles, and each coda waveform was compared with the waveform recorded during the preceding fatigue interval. By restricting the analysis to consecutive material states, waveform correlation was preserved throughout fatigue loading, allowing small velocity perturbations to be quantified without the progressive decorrelation associated with comparisons to a single pristine baseline.

Let *s_k_*_−1_(*t*) and *s_k_*(*t*) denote the coda waveforms corresponding to two consecutive material states. The stretching method determines the temporal dilation that maximizes the normalized cross-correlation between the two waveforms,

(1) $$C_{r}\left(\alpha \right)=\frac{\int_{t-T/2}^{t+T/2}s_{k}[t(1+\alpha )]\;s_{k-1}(t)\;\mathrm{dt}}{\sqrt{\int_{t-T/2}^{t+T/2}s_{k}^{\;2}[t(1+\alpha )]\;\mathrm{dt}\int_{t-T/2}^{t+T/2}s_{k-1}^{\;2}(t)\;\mathrm{dt}}}$$

The optimum stretching parameter is obtained from

(2) $$\alpha_{k}=\underset{\alpha \in \Omega }{\mathrm{arg max}}\left[C_{r}(\alpha )\right]$$

where *T* is the selected coda-wave window and Ω is the prescribed search interval for the stretching parameter. The parameter *α_k_* represents the average relative velocity variation between two consecutive material states.

**Figure 6:**
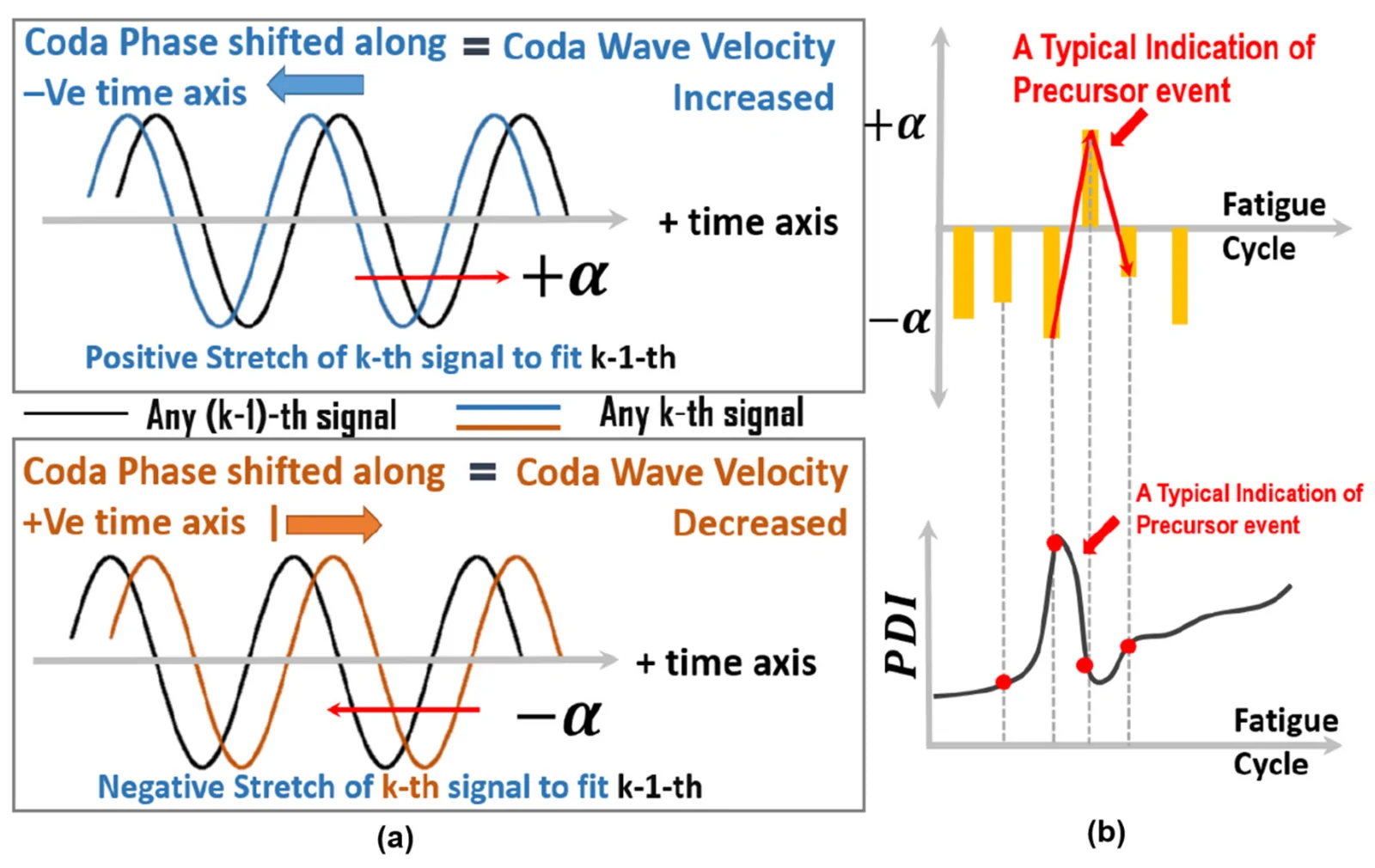
Conceptual representation of incremental coda-wave interferometry: (**a**) positive and negative stretch parameters associated with changes in coda-wave velocity between consecutive material states and (**b**) accumulation of the incremental stretch parameters to construct the Precursor Damage Index (PDI). Adapted from [9].

The incremental stretch parameters obtained after each 5000-cycle fatigue interval were subsequently accumulated to establish the Precursor Damage Index (PDI),

(3) $$\mathrm{PDI}=\left|\sum_{k=1}^{N}\alpha_{k}\right|$$

which provides a continuous measure of material-state progression throughout fatigue loading. Unlike CWI approaches that compare all measurements with a single pristine reference, the incremental framework adopted in this study compared consecutive material states, preserving waveform correlation while continuously tracking fatigue-induced material changes. Figure 6 illustrates the extraction of positive and negative incremental stretch parameters and their accumulation to construct the PDI.

### 4.3. Experimental Observations During Incremental Fatigue Loading

Incremental guided-wave measurements were performed after every 5000 fatigue cycles up to 300,000 cycles, corresponding to the precursor fatigue regime before the initiation of visible delamination. Comparison of the first-arriving Lamb-wave packets revealed only minor variations in waveform morphology throughout the loading history. The direct guided-wave response remained highly consistent between successive fatigue states, indicating that the accumulated fatigue damage produced only limited changes in the effective propagation characteristics of the dominant Lamb-wave modes.

In contrast, systematic temporal shifts developed within the coda-wave portion of the guided-wave response as fatigue progressed. Although consecutive coda waveforms remained strongly correlated, application of the stretching procedure identified measurable relative velocity variations that increased progressively with fatigue loading. The preserved waveform correlation confirmed the suitability of the incremental comparison strategy, while the extracted stretching parameters demonstrated that the scattered wavefield was considerably more sensitive to fatigue-induced material-state changes than the direct wave packets.

**Figure 7:**
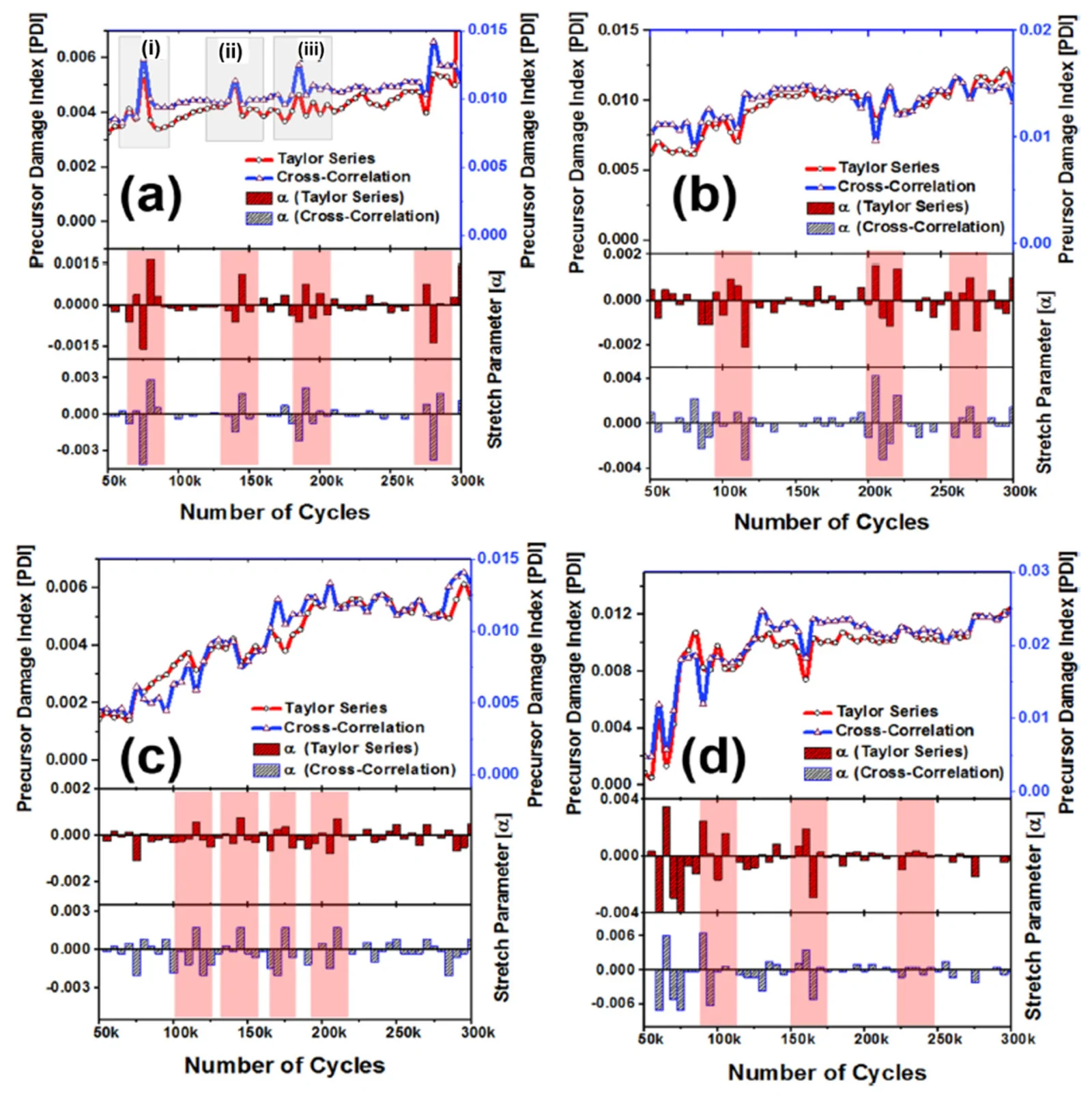
Incremental stretch parameter and Precursor Damage Index (PDI) measured during fatigue loading of CFRP specimens, (**a**) S-A, (**b**) S-B, (**c**) S-C, and (**d**) S-D. The accumulated PDI tracks progressive material-state changes and identifies precursor-damage activity beginning at approximately 120,000–160,000 cycles. Adapted from [9].

The incremental stretch parameters were subsequently accumulated to construct the Precursor Damage Index (PDI), which exhibited continuous evolution throughout the investigated fatigue range, as shown in Figure 7. Rather than indicating abrupt damage initiation, the PDI reflected the gradual accumulation of distributed precursor damage prior to the formation of macroscopic fatigue features. The continuous progression of the PDI is consistent with the distributed nature of fatigue degradation in woven CFRP laminates, where matrix cracking and fiber–matrix interfacial damage develop progressively before visible delamination becomes apparent.

The comparison between the direct guided-wave response and the coda-wave analysis demonstrates the principal contribution of guided CWI. While conventional guided-wave features remained largely unchanged during the investigated fatigue regime, the coda-wave analysis provided a continuous measure of fatigue-induced material-state evolution through incremental relative velocity variations. These observations establish guided CWI as an effective approach for monitoring distributed precursor damage before the development of visible fatigue defects and provide the basis for comparison with the nonlinear ultrasonic techniques discussed in the following sections.

### 4.4. Implications of Coda Wave Assessment

The enhanced sensitivity of guided coda-wave interferometry arises from the cumulative interaction of the coda wave with the evolving heterogeneous microstructure of the laminate. During precursor fatigue loading, distributed matrix cracking, fiber–matrix interfacial degradation, and local stiffness variations progressively modify the elastic properties along numerous scattering paths. Although these perturbations produce only minor changes in the first-arriving Lamb-wave packets, their cumulative influence becomes measurable within the multiply scattered coda wave through incremental relative velocity variations. The resulting Precursor Damage Index therefore provides a quantitative measure of material-state evolution before the development of visible fatigue damage.

While guided CWI demonstrated substantially greater sensitivity than conventional guided-wave measurements, it remains an indirect indicator of microstructural evolution because the measured velocity variations represent the cumulative response of the propagating wavefield rather than individual damage mechanisms [33,34]. Consequently, guided CWI is particularly effective for monitoring the progression of distributed precursor damage but cannot independently distinguish the specific physical processes responsible for the observed material-state changes. This limitation motivates the complementary nonlinear ultrasonic approaches presented in the following sections, where nonlinear wave interactions provide additional sensitivity to the underlying mechanisms governing early-stage fatigue damage.

## 5. High-Frequency Ultrasonic Pulse-Echo Assessment of Precursor Damage

### 5.1. Physical Basis of High-Frequency Pulse-Echo Ultrasonics

Guided coda-wave interferometry provides a sensitive measure of progressive material-state changes by tracking cumulative variations in wave propagation. However, the coda-wave response represents the integrated effect of distributed scattering along the propagation path and does not independently describe the associated nonlinear material response. High-frequency pulse-echo ultrasonics was therefore employed as a complementary method for examining fatigue-induced acoustic nonlinearity within localized regions of the composite. This transition from guided-wave interferometry to localized through-thickness interrogation extends the assessment from distributed material-state monitoring to spatially resolved characterization of nonlinear ultrasonic behavior.

In an ideal linear elastic medium, a finite-amplitude ultrasonic wave retains its original spectral composition during propagation. Fatigue-induced matrix cracking, fiber–matrix interfacial degradation, local debonding, and micro-void formation introduce weak nonlinearities into the stress–strain response. Interaction of the propagating wave with these features produces waveform distortion, higher-order harmonics, and sideband modulation that can be more sensitive to microstructural degradation than conventional linear ultrasonic parameters [21,22,23,35].

**Figure 8:**
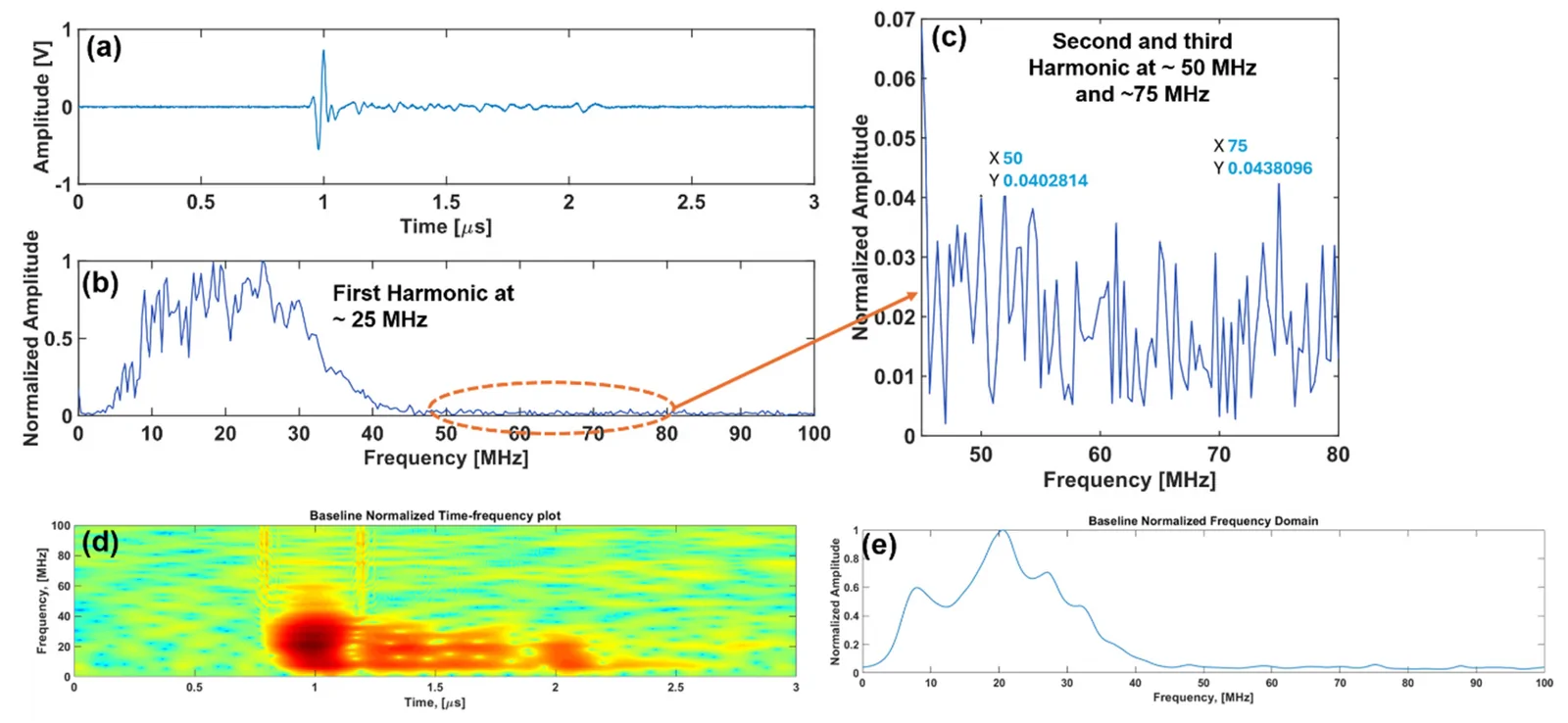
Processing of the high-frequency pulse-echo response for nonlinear parameter extraction: (**a**) selected time-domain signal containing the relevant surface reflections, (**b**) FFT of time-domain signal showing first harmonic frequency at ~25 MHz, (**c**) Zoom in part of FFT signal showing second and third harmonic frequencies at ~50 MHz and at ~75 MHz, (**d**) baseline normalized time-frequency representation, (**e**) frequency-domain spectrum after baseline normalization with enhanced peak separation. Adapted from [30].

The present approach employed a 25 MHz longitudinal P-wave in immersion pulse-echo mode to interrogate the laminate through its thickness. The shorter wavelength associated with high-frequency P-waves increases sensitivity to localized material heterogeneity, while spatial scanning permits the nonlinear response to be mapped across the examined specimen region. Rather than relying solely on travel time or reflected amplitude, the analysis quantified precursor damage using second- and third-order nonlinear parameters and sideband peak modulation extracted through the processing sequence shown in Figure 8 [30].

### 5.2. Nonlinear Parameter and Sideband Extraction

The recorded pulse-echo waveform contained reflections from the front and back surfaces of the laminate. Because the nonlinear response was encoded primarily within its spectral composition, the portion of the waveform containing these reflections was retained and transformed into the frequency domain using the fast Fourier transform. Complex Morlet wavelet convolution was subsequently applied for time–frequency analysis and baseline normalization. This procedure suppressed acquisition-related artifacts while preserving the spectral features associated with material nonlinearity.

**Figure 9:**
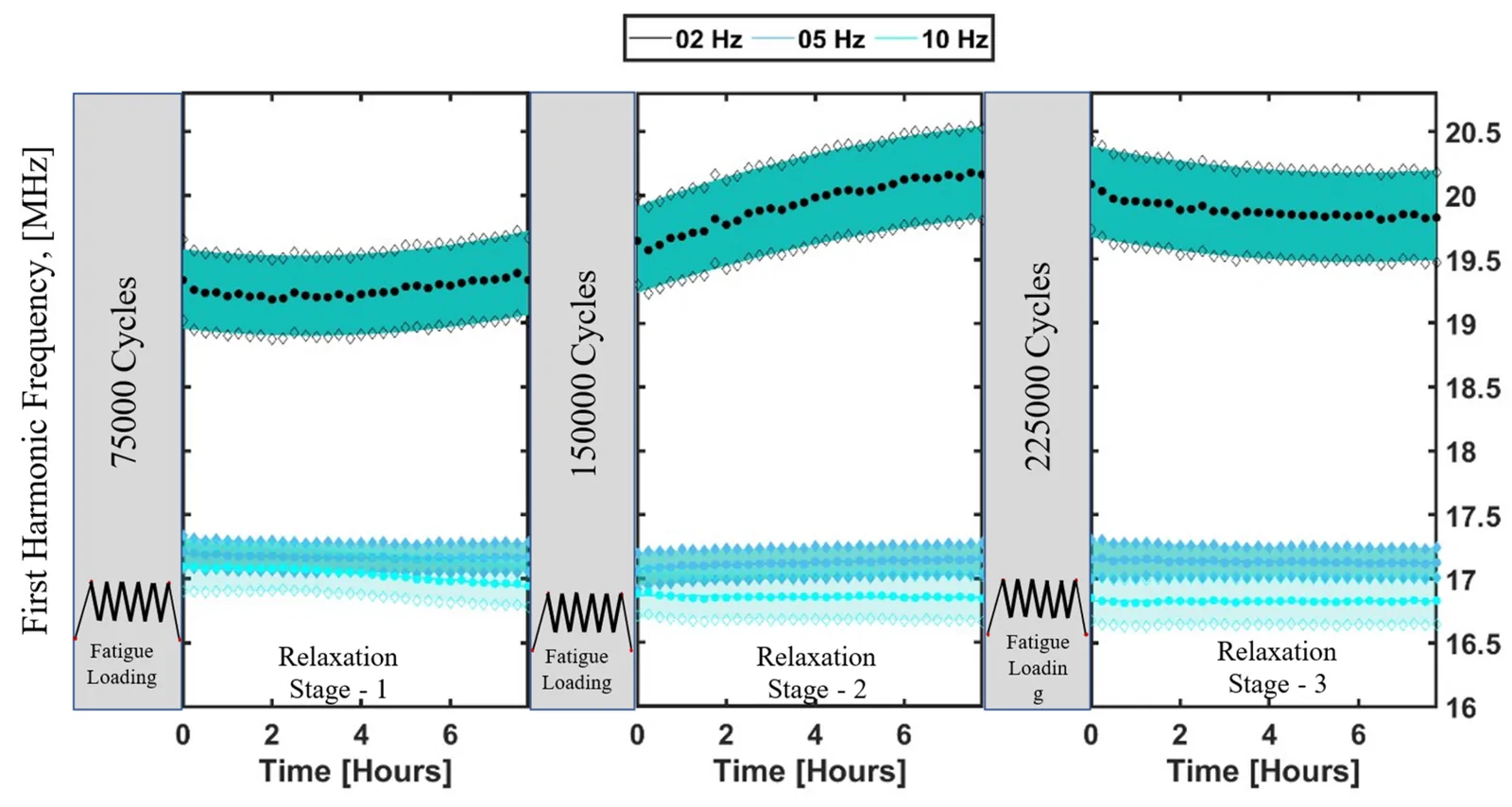
Mean fundamental frequency with tolerance (*μ* ± 0.1*σ*) during the 8 h relaxation periods following successive fatigue stages for CFRP specimens loaded at 2, 5, and 10 Hz. Adapted from [30].

**Figure 10:**
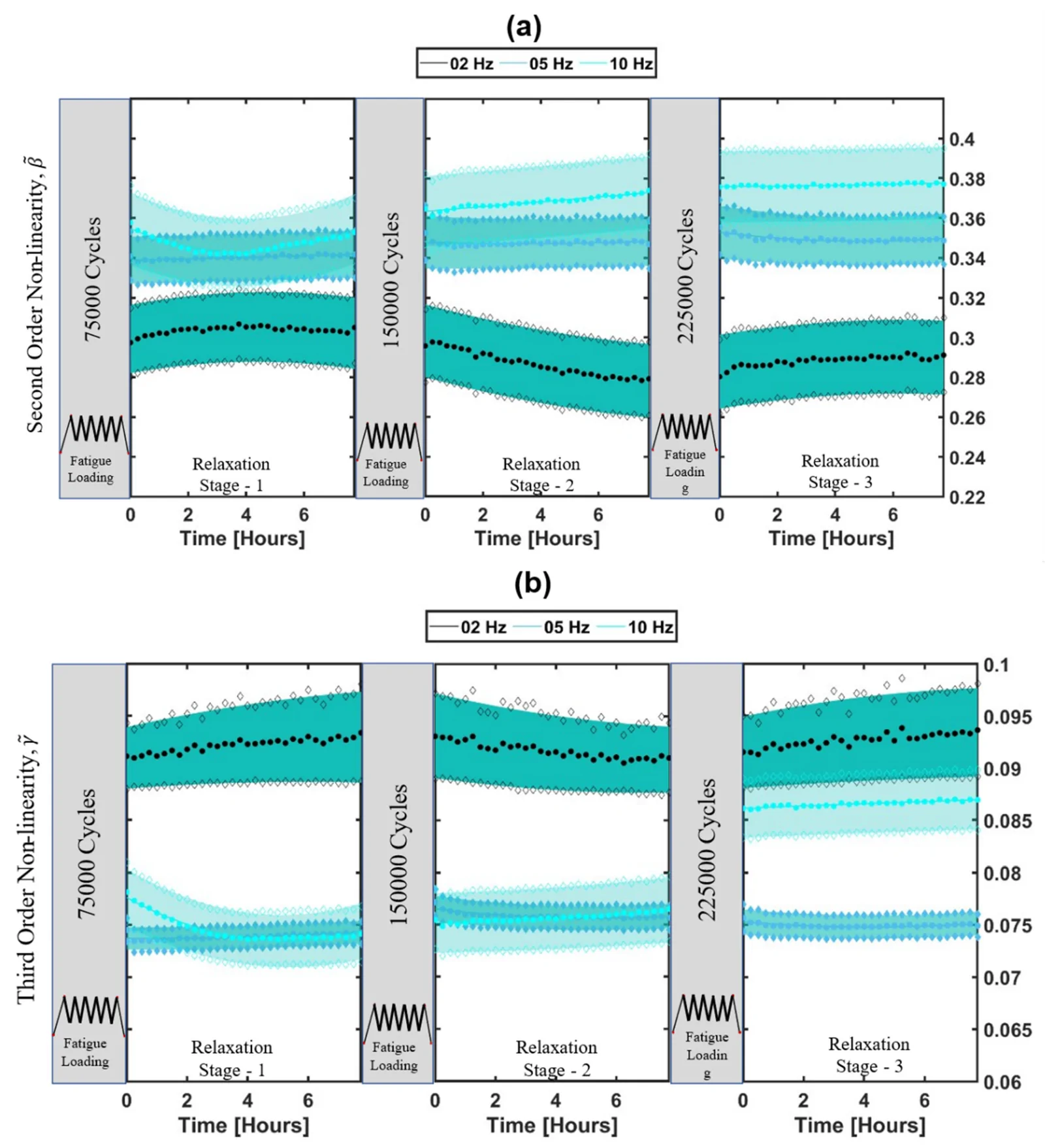
Changes in the nonlinear ultrasonic parameters with tolerance (*μ* ± 0.1*σ*) during the three fatigue relaxation stages for specimens loaded at 2, 5, and 10 Hz: (**a**) normalized second-order parameter, $\tilde{\beta }$, and (**b**) normalized third-order parameter, $\tilde{\gamma }$. Adapted from [30].

The baseline-normalized spectrum was used to identify the fundamental amplitude, *A*_1_, and the corresponding second- and third-harmonic amplitudes, *A*_2_ and *A*_3_. A frequency tolerance of ±1 MHz around the expected harmonic frequencies was used to accommodate minor spectral drift while limiting the influence of isolated noise peaks. The harmonic amplitudes were then used to calculate the normalized second- and third-order nonlinear parameters:

(4) $$\tilde{\beta }=\frac{A_{2}}{A_{1}^{2}}$$

(5) $$\tilde{\gamma }=\frac{A_{3}}{A_{1}^{3}}$$

where *A*_1_, *A*_2_, and *A*_3_ denote the amplitudes of the fundamental, second, and third harmonics, respectively. Under consistent acquisition conditions, these normalized ratios reduce dependence on excitation amplitude and permit comparison among fatigue states and loading frequencies. The parameters were calculated at approximately 9375 spatial locations during each measurement interval. The resulting datasets were fitted using statistical distributions selected through the Bayesian Information Criterion before their representative mean values and variations were determined [29]. The fundamental-frequency and nonlinear-parameter results are presented in Figure 9 and Figure 10, respectively.

**Figure 11:**
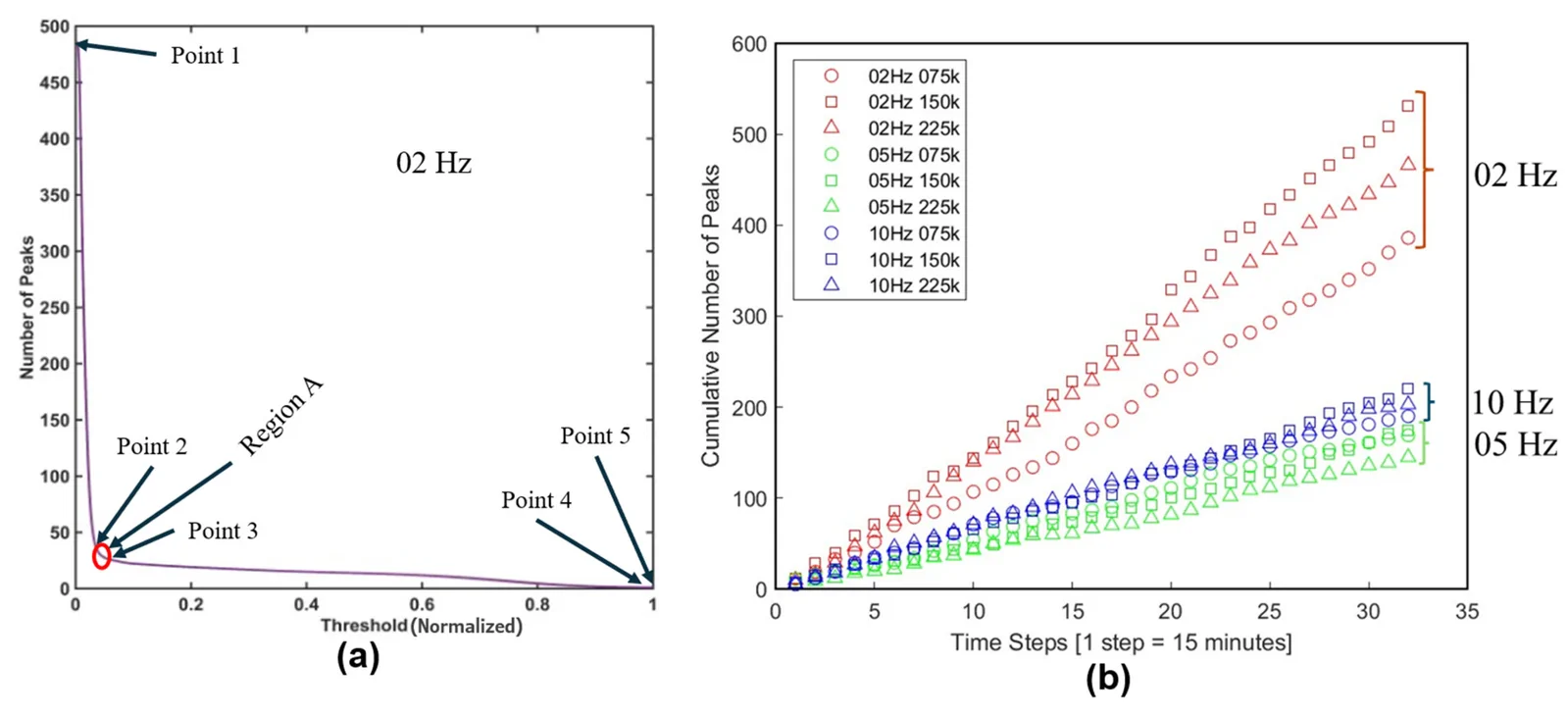
Sideband peak analysis of the high-frequency pulse-echo signals: (**a**) variation in the number of detected spectral peaks with the normalized amplitude threshold and (**b**) cumulative sideband peak counts for specimens loaded at 2, 5, and 10 Hz from the pristine condition through the three fatigue relaxation stages. The 5 Hz specimens exhibited the lowest cumulative peak count after 225,000 cycles, indicating the greatest precursor damage. Adapted from [30].

Sideband peaks were identified by counting local spectral maxima above a normalized amplitude threshold. The threshold was selected from the geometric transition in the peak count versus normalized threshold curve, not by maximizing separation among the loading conditions. At thresholds approaching 0%, nearly all local spectral fluctuations were counted, whereas thresholds approaching 100% retained only the dominant spectral peak. Between these limits, the curve changed from a steep descending slope to a shallow descending slope, defining the transition region identified as Region A in Figure 11a. Point 2, corresponding to approximately 5% of the maximum spectral amplitude, was selected because it lies within this transition region and retains more spectral peaks than the higher threshold represented by Point 3. Examination of the archived curves confirmed that a comparable transition occurred for the 2, 5, and 10 Hz conditions. The same 5% threshold was therefore applied to all loading frequencies without condition-specific adjustment. In addition to the peak count evaluated at the individual 5% threshold, the cumulative sideband peak count, N_SB_, was calculated over the selected threshold range within Region A. This cumulative measure incorporates the peak counts obtained across multiple threshold levels and reduces reliance on the result at any single threshold. It was used to compare spectral modulation across loading frequencies, fatigue stages, and relaxation intervals. Unlike $\tilde{\beta }$ and $\tilde{\gamma }$, which are calculated from amplitudes at specified harmonic frequencies, N_SB_ represents spectral redistribution across a broader frequency range.

A sensitivity check was also performed at individual thresholds of 2%, 5%, and 10%. After 225,000 cycles, the mean peak counts for the 2, 5, and 10 Hz groups were 195.82, 124.46, and 139.03, respectively, at 2%; 34.04, 28.07, and 27.51 at 5%; and 27.94, 23.05, and 22.62 at 10%. The 2 Hz group retained the highest mean peak count at all three thresholds. However, the relative ordering of the 5 and 10 Hz groups changed with threshold: the 5 Hz count was lower at 2%, whereas the 10 Hz count was slightly lower at 5% and 10%. The individual-threshold result therefore provides a robust distinction between the 2 Hz group and the higher-frequency groups but does not independently establish the relative ranking of the 5 and 10 Hz groups. Their relative damage states were interpreted using the cumulative *N_SB_* response together with the harmonic parameters and convergent mechanical and microstructural evidence.

### 5.3. Experimental Observations During Fatigue Loading

Figure 9 presents the mean fundamental frequencies measured during the three post-fatigue relaxation stages for specimens loaded at 2, 5, and 10 Hz. High-frequency pulse-echo measurements were performed during the 8 h unloaded period following each block of 75,000 fatigue cycles. The first harmonic frequency exhibited relatively limited variation compared with the nonlinear parameters. Across the three relaxation stages, the mean first harmonic frequencies remained within approximately 19.25–20.25 MHz for the 2 Hz specimens, 17.00–17.25 MHz for the 5 Hz specimens, and 16.80–17.10 MHz for the 10 Hz specimens. Although these differences reflected loading-rate-dependent changes in the ultrasonic response, the fundamental component did not provide a consistent measure of progressive precursor damage across all fatigue stages.

As shown in Figure 10a, the normalized second-order parameter, $\tilde{\beta }$, exhibited an overall increase with accumulated fatigue loading. During the first relaxation stage, $\tilde{\beta }$ increased steadily for the 2 and 5 Hz specimens, whereas the 10 Hz specimens showed an initial reduction followed by a gradual increase. An upward trend persisted for the 5 and 10 Hz specimens during the second stage, and $\tilde{\beta }$ increased for all loading frequencies during the third stage. The pronounced response at the higher loading frequencies indicates that second-order nonlinearity became progressively more sensitive as interfacial degradation accumulated. This behavior is consistent with an increasing contribution from interfacial sliding, crack-edge interaction, and fiber–matrix debonding, although the individual contributions cannot be separated from the present measurements.

In contrast, the normalized third-order parameter, $\tilde{\gamma }$, followed an opposing trend, as shown in Figure 10b. Its values generally decreased as fatigue damage accumulated, a trend consistent with a reduction in the effective cubic nonlinear response of the polymer matrix as microcracking and other forms of distributed degradation developed. The 2 Hz specimens retained higher $\tilde{\gamma }$ values throughout the three stages, consistent with the lower degradation identified by the accompanying measurements. After 225,000 cycles, the 5 Hz specimens exhibited lower $\tilde{\gamma }$ values than the 10 Hz specimens, identifying the 5 Hz condition as the most degraded material state. The opposing responses of $\tilde{\beta }$ and $\tilde{\gamma }$ therefore provide complementary indicators: increasing $\tilde{\beta }$ is consistent with a growing contribution from interface-dominated nonlinearity, whereas decreasing $\tilde{\gamma }$ is consistent with degradation of the matrix-dominated nonlinear response.

Sideband peak analysis presented in Figure 11 provided an independent description of spectral changes during fatigue loading. The 2 Hz specimens generally retained the largest cumulative peak counts, corresponding to the least degraded material state. In contrast, the 5 Hz specimens exhibited the lowest cumulative peak count after 225,000 cycles, identifying this loading condition as the most severely affected. The 10 Hz specimens exhibited an intermediate response. These loading-rate-dependent trends agreed with those obtained from the harmonic parameters despite the different basis of the sideband analysis.

Correlation analysis during the third relaxation stage further demonstrated consistency among the nonlinear indicators. The cumulative sideband peak count exhibited a strong negative correlation with $\tilde{\beta }$, with *r* ≈ −0.98, and a strong positive correlation with $\tilde{\gamma }$, with *r* ≈ +0.95. Thus, the experimentally identified progression of precursor damage was associated with increasing $\tilde{\beta }$, decreasing $\tilde{\gamma }$, and decreasing *N*_SB_. The agreement among these complementary spectral indicators reduces the likelihood that the observed trends resulted solely from changes in signal amplitude, instrumentation, or processing. Their relationships with residual strength and observed microstructural degradation are evaluated independently in Section 8.

### 5.4. Implications of High-Frequency Pulse-Echo Assessment

High-frequency pulse-echo measurements provided localized variations in nonlinear material response and sensor-independent characterization of through-thickness material response. The combined behavior of $\tilde{\beta }$, $\tilde{\gamma }$, and *N*_SB_ showed that precursor degradation altered several aspects of the measured spectrum rather than a single harmonic component. Spatial scanning further allowed these changes to be assessed across the specimen region.

The method is better suited to targeted inspection than continuous monitoring because CFRP attenuation restricts propagation distance and immersion scanning requires controlled coupling and surface access [36]. Its principal contribution to the framework is therefore localized confirmation of material changes detected through structural-scale guided wave measurements.

## 6. Guided-Wave Nonlinear Ultrasonic Assessment

### 6.1. Physical Basis

High-frequency pulse-echo measurements provide localized through-thickness characterization but require point-by-point scanning and are limited by attenuation over extended propagation distances. Guided Lamb waves provide a complementary approach because they propagate over substantially larger regions of plate-like structures and can be generated and received using permanently bonded piezoelectric sensors. This capability makes them suitable for structural-scale monitoring without requiring full-field scanning.

The first-arriving guided-wave packet, however, exhibited only limited changes during the investigated precursor regime. This is consistent with the observations presented in Section 4, where conventional amplitude and arrival-time features remained comparatively insensitive to distributed early-stage degradation. Additional information can be obtained from the nonlinear spectral components generated as the guided wave interacts with the evolving laminate [37,38]. Matrix microcracking, fiber–matrix separation, interfacial sliding, and local crack-edge contact distort the propagating waveform and transfer energy from the fundamental component to higher harmonics. These higher-order components may change substantially before the fundamental guided-wave packet exhibits a readily measurable variation.

The guided-wave nonlinear investigation used a fundamental *S*_0_ mode and a second harmonic *S*_0_ mode selected to provide approximate phase matching at a relatively low excitation frequency. The selected mode and frequency provided sufficient propagation distance for structural-scale interrogation while retaining sensitivity to fatigue-induced nonlinear interactions. The method therefore examined a different part of the guided-wave response from CWI. CWI quantified phase variations within the multiply scattered coda, whereas guided-wave nonlinear ultrasonics quantified higher-harmonic generation associated with the direct propagating wavefield [27,28].

### 6.2. Signal Processing

Guided waves were generated using a five-cycle tone burst with a central frequency of 330 kHz and an excitation amplitude of 20 V peak-to-peak. The actuator and receiver were bonded to the specimen surface with a center-to-center separation of 90 mm. At each measurement interval, a 512-point moving-average filter was applied to the recorded waveform to smooth rapid fluctuations and reduce high-frequency noise. The piezoelectric sensor capacitance was monitored throughout the experiment to identify potential changes in sensor condition.

Before fatigue testing, the contribution from the measurement system was evaluated by monitoring a pristine specimen at 15 min intervals. The measured variation in acoustic nonlinearity remained below approximately 0.1%, indicating that the changes subsequently observed in the fatigued specimens exceeded the background variability of the instrumentation. This baseline test was particularly important because higher-harmonic measurements can be influenced by the signal generator, amplifier, piezoelectric elements, adhesive layers, and data-acquisition system.

The processed time-domain signals were transformed into the frequency domain using the fast Fourier transform. The measured fundamental component occurred at approximately 320 kHz, close to the 330 kHz excitation frequency. The amplitudes of the fundamental, second harmonic, and third harmonic were denoted by *A*_1_, *A*_2_, and *A*_3_, respectively. The normalized second- and third-order parameters, $\tilde{\beta }$ and $\tilde{\gamma }$, were calculated using the amplitude ratios defined in Equations (4) and (5). Applying the same normalized formulation used for the high-frequency P-waves allowed the nonlinear responses of the two propagation configurations to be compared on a common basis.

For fatigue-state comparison, the parameter values measured immediately after each 75,000-cycle loading block were used to characterize the unrelaxed material states. The resulting second- and third-order quantities were expressed as nonlinear damage index parameters and compared with the pristine response. Measurements obtained after the subsequent 8 h unloaded period were also calculated, but their time-dependent changes are examined separately in Section 7. This separation prevents the progression of fatigue-induced nonlinearity from being conflated with its reduction during post-fatigue stress relaxation.

### 6.3. Experimental Observations

**Figure 12:**
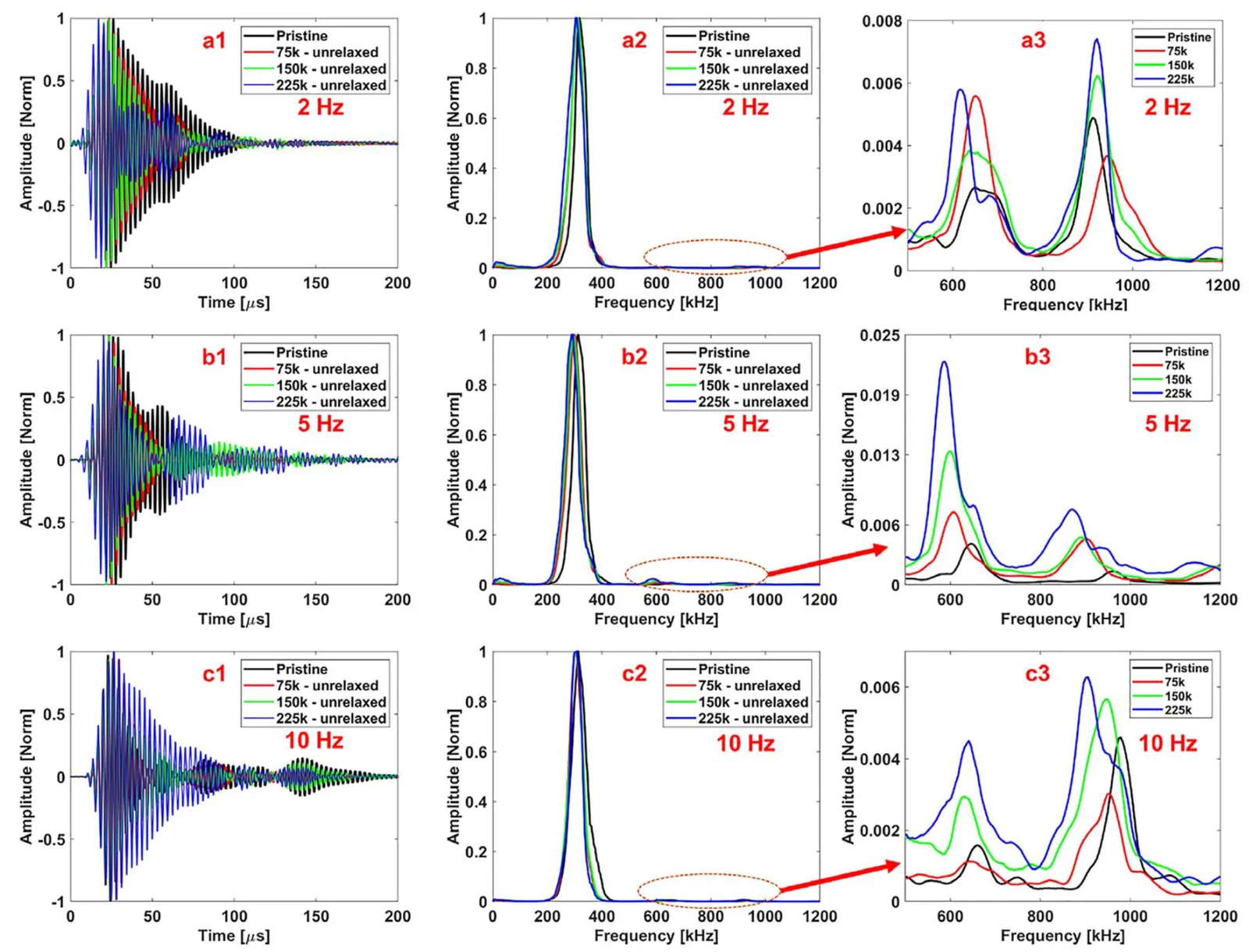
Guided-wave responses used for higher-order nonlinear analysis of specimens loaded at 2, 5, and 10 Hz: (**a1**–**c1**) time-domain signals obtained in the pristine state and immediately after 75,000, 150,000, and 225,000 fatigue cycles; (**a2**–**c2**) corresponding frequency spectra; and (**a3**–**c3**) enlarged spectral regions containing the second- and third-harmonic components. Adapted from [28].

As shown in Figure 12, the time-domain guided-wave signals showed only minor changes across the three fatigue stages, whereas the corresponding spectra revealed measurable changes in the higher-harmonic components. The first-arriving wave packets remained similar in amplitude and morphology, confirming that conventional linear features were weakly affected during the investigated precursor regime.

The second- and third-harmonic amplitudes generally increased relative to the pristine state as fatigue loading accumulated. For the 5 and 10 Hz specimens, the harmonic amplitudes after 225,000 cycles exceeded those measured after 150,000 cycles, which in turn generally exceeded those measured after 75,000 cycles. The harmonic components also shifted toward lower frequencies with increasing fatigue exposure. The combined increase in amplitude and reduction in harmonic frequency indicated progressive modification of the nonlinear and effective elastic response of the laminate.

The 2 Hz specimens produced a less uniform response during the earlier fatigue stages. An increase in third-harmonic amplitude and a shift toward lower frequency were evident, while the corresponding second-harmonic trend became clearer after 150,000 and 225,000 cycles. This behavior indicates that the sensitivity of individual harmonic parameters depends on both the accumulated material state and the loading condition. It also demonstrates why a single harmonic measurement should not be treated as a universal indicator across all stages of precursor damage.

**Figure 13:**
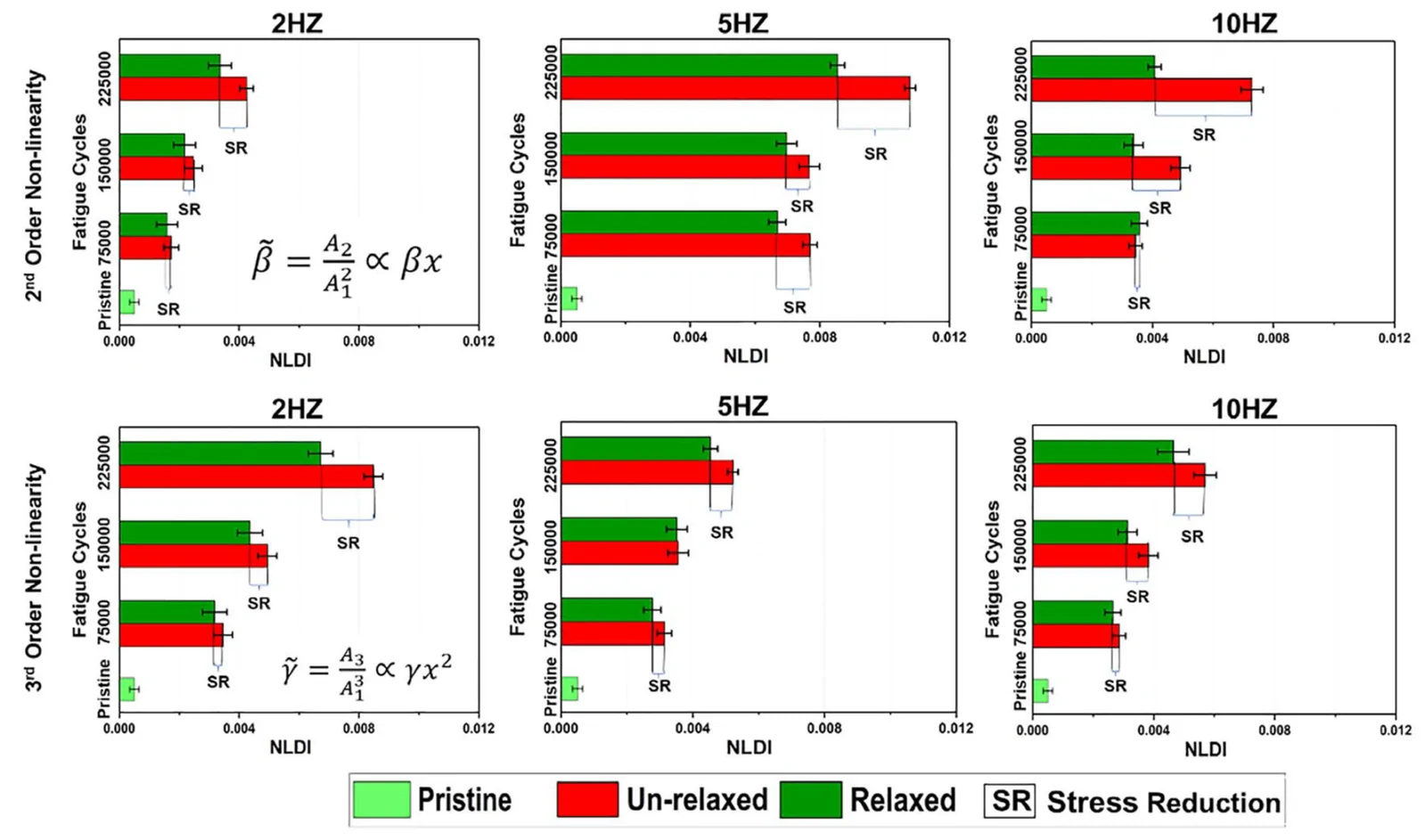
Second- and third-order nonlinear damage indices obtained from guided-wave measurements of specimens loaded at 2, 5, and 10 Hz. The unrelaxed values were measured immediately after 75,000, 150,000, and 225,000 fatigue cycles, while the relaxed values were measured after the corresponding 8 h unloaded periods. The difference between each pair represents the reduction in nonlinear response during stress relaxation. Adapted from [28].

The normalized nonlinear parameters provided a clearer comparison among the three loading frequencies. The second-order nonlinear damage index, based on $\tilde{\beta }$, generally increased with accumulated fatigue cycles. The 5 Hz specimens exhibited higher values than the 2 and 10 Hz specimens after 75,000, 150,000, and 225,000 cycles, identifying the 5 Hz condition as the most severely degraded of the three loading cases. The third-order parameter also changed relative to the pristine state, although its progression was not strictly monotonic under every combination of loading frequency and fatigue stage. The two parameters should therefore be interpreted as complementary measures of the nonlinear guided-wave response rather than interchangeable damage indices. Figure 13 presents both the unrelaxed and relaxed nonlinear damage indices. The unrelaxed values are considered here as indicators of accumulated fatigue degradation, whereas the reductions measured after the 8 h unloaded periods are examined in Section 7.

The loading-frequency dependence was not monotonic. Although increasing fatigue frequency can increase the rate of cyclic energy input, damage accumulation also depends on the time available for viscoelastic deformation and stress redistribution during each cycle. Within the tested range, the intermediate loading frequency produced the largest nonlinear response. This ranking was subsequently supported by high-frequency P-wave measurements and by the convergent mechanical and microscopic evidence from the coordinated Route II dataset presented in Section 8. This frequency ranking applies to the woven CFRP material system, loading amplitude, specimen geometry, and fatigue protocol investigated here. It should not be interpreted as evidence that 5 Hz generally produces greater fatigue degradation than other loading frequencies in CFRP composites.

The agreement between guided-wave and high-frequency nonlinear measurements is important because the two techniques used different propagation paths and sensing configurations. Guided waves sampled an extended in-plane region using bonded sensors, whereas the P-waves interrogated localized regions through the laminate thickness without bonded sensors. Their consistent identification of the 5 Hz specimens as the most degraded supports the interpretation that the measured nonlinear changes originated predominantly from the fatigue-affected composite rather than from a particular sensing configuration [30].

### 6.4. Implications of Guided-Wave Nonlinear Assessment

Guided wave nonlinear analysis extended precursor damage assessment over the actuator and receiver path using permanently bonded sensors. Changes in higher harmonic components provided information that was not apparent from the morphology of the direct wave packet, supporting structural-scale monitoring without spatial scanning.

The measured nonlinearity may include contributions from the excitation system, PZT sensors, adhesive layers, mode selection, and environmental conditions. Guided wave nonlinear parameters should therefore be interpreted through controlled baselines, sensor integrity measurements, and standardized acquisition conditions. Their dependence on post-fatigue measurement time is examined in Section 7.

## 7. Influence of Stress Relaxation on Ultrasonic Damage Assessment

### 7.1. Physical Basis of Stress Relaxation in CFRP

The ultrasonic response of a fatigue-loaded polymer composite depends not only on accumulated microstructural damage but also on its time-dependent mechanical state. During cyclic loading, the viscoelastic matrix undergoes time-dependent deformation while heterogeneous stress transfer among the fibers, matrix, and interfaces produces localized residual stresses. When the applied load is removed, part of this internal stress state gradually redistributes, producing partial recovery of the effective stiffness and a corresponding change in ultrasonic wave propagation. Measurements obtained immediately after fatigue loading may therefore contain contributions from both persistent damage-related material response and recoverable stress relaxation.

These contributions affect nonlinear ultrasonic behavior differently over time. Matrix cracking, fiber–matrix debonding, interfacial sliding, and isolated fiber damage accumulate across successive fatigue stages and produce a persistent increase in acoustic nonlinearity. In contrast, the contribution associated with the transient internal stress state decreases during unloaded relaxation. This reduction appears as a decrease in second-harmonic amplitude and a partial return of the harmonic frequency toward its preloading position. The nonlinear response measured at a given fatigue state can therefore be considered a combination of a persistent component associated predominantly with accumulated damage and a dissipative component associated with time-dependent stress redistribution.

The distinction is important because an ultrasonic measurement obtained at a single post-loading time cannot determine whether an elevated nonlinear response originates from persistent damage-related change or from the temporary mechanical state of the composite. Repeated measurements during a controlled unloaded period were therefore used to examine how the ultrasonic response approached a more stable condition following each fatigue stage. This time-resolved approach provided the basis for separating the progressive growth of damage-related nonlinearity from the portion dissipated during stress relaxation [27,28].

### 7.2. Separation of Dissipative and Persistent Nonlinearity

Guided Lamb-wave measurements were collected every 15 min during the 8 h unloaded period following each block of 75,000 fatigue cycles. A five-cycle tone burst with a central frequency of 330 kHz and an excitation amplitude of 20 V peak-to-peak was applied through the bonded piezoelectric actuator. A 512-point moving-average filter was applied to the recorded waveforms before they were transformed into the frequency domain. The fundamental and second-harmonic amplitudes were then used to calculate the normalized second-order parameter, $\tilde{\beta }$, following the formulation introduced previously.

Two values were used to characterize the nonlinear response following each fatigue stage. The value obtained immediately after removal from the testing machine, ${\tilde{\beta }}_{0}$, represented the unrelaxed material state. The value obtained after 8 h, ${\tilde{\beta }}_{8h}$, represented the relaxed state under the prescribed experimental conditions. The relative reduction in second-order nonlinearity during relaxation was quantified as

(6) $$R_{\tilde{\beta }}=\frac{{\tilde{\beta }}_{0}-{\tilde{\beta }}_{8h}}{{\tilde{\beta }}_{0}}\times 100\%$$

where $R_{\tilde{\beta }}$ represents the dissipated portion of the nonlinear response during the 8 h unloaded period. The value retained after relaxation, ${\tilde{\beta }}_{8h}$, was used to compare the persistent nonlinear response among successive fatigue stages. A pristine specimen monitored for 24 h exhibited less than approximately 0.1% variation in acoustic nonlinearity, indicating that the measured time-dependent changes were substantially greater than the background variability of the instrumentation.

The companion pulse-echo measurements provided an independent assessment of the relaxation response without bonded sensors. At each scanned location, the back-surface reflection amplitude was divided by the front-surface reflection amplitude. The spatial mean of this ratio, referred to as the mean amplitude ratio, was tracked during the same 8 h unloaded period. Because internal damage reduced through-thickness wave transmission, lower initial amplitude ratios indicated a more degraded material state. Subsequent increases during relaxation were interpreted as evidence of partial recovery associated with internal stress redistribution [28].

### 7.3. Experimental Observations During Post-Fatigue Relaxation

**Figure 14:**
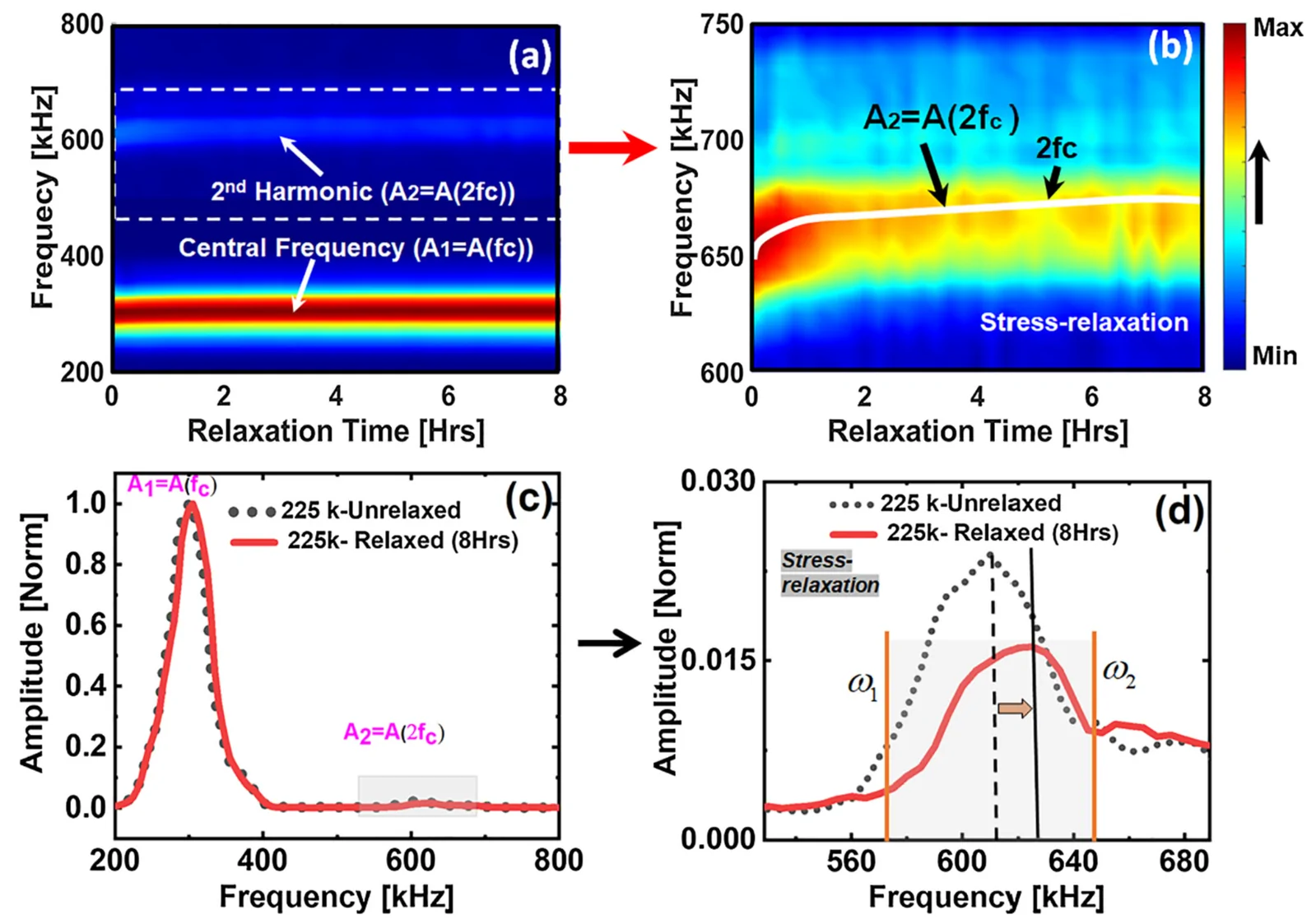
Spectral changes in the guided-wave response during the 8 h relaxation period following 225,000 fatigue cycles: (**a**) frequency response as a function of relaxation time, (**b**) enlarged second-harmonic region, (**c**) comparison of the unrelaxed and 8 h relaxed spectra, and (**d**) corresponding second-harmonic amplitudes. The reduction in second-harmonic amplitude and its partial shift toward a higher frequency demonstrate dissipation of the transient nonlinear response during relaxation. Adapted from [27].

**Figure 15:**
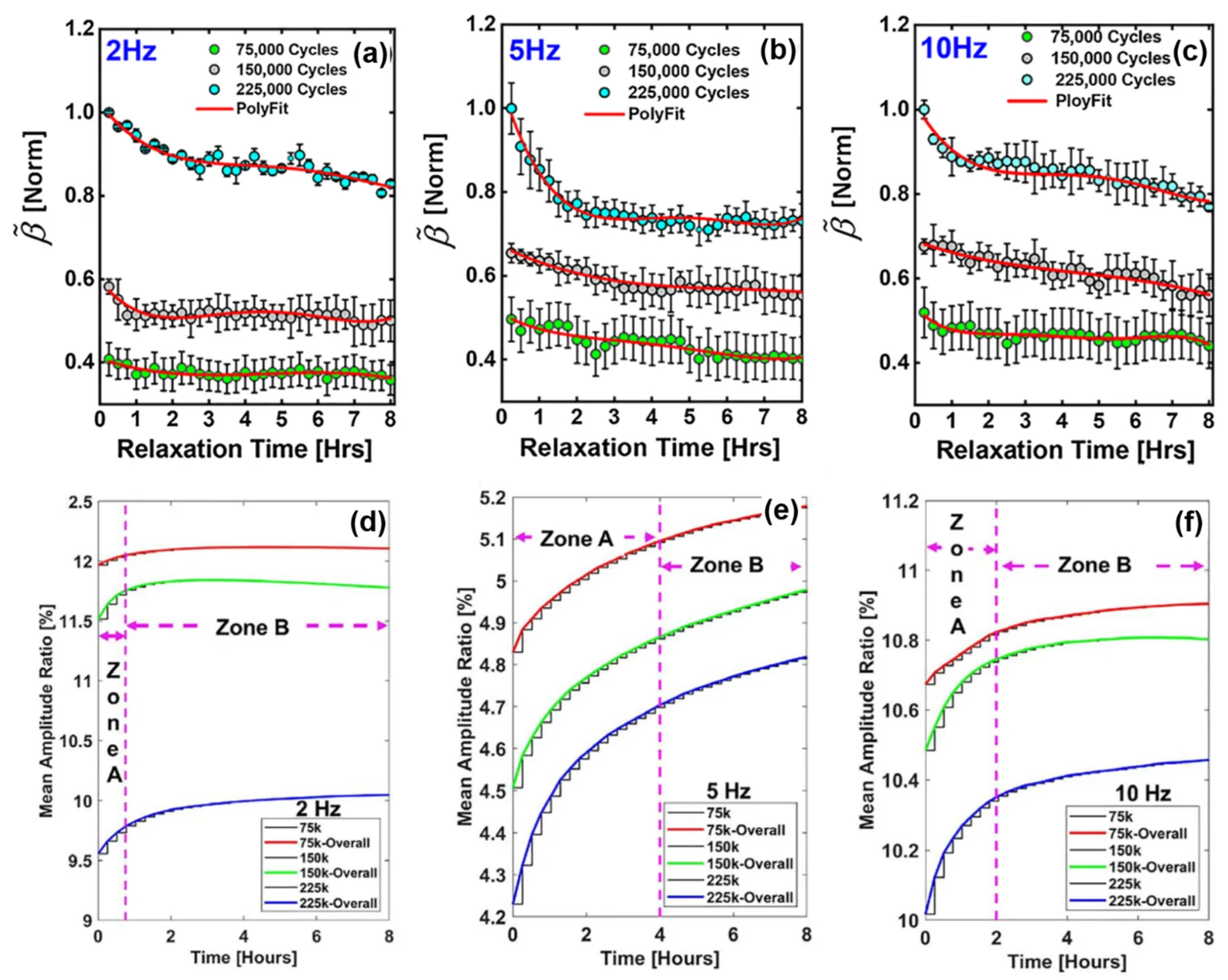
Time-dependent ultrasonic responses during the 8 h unloaded periods following successive fatigue stages: (**a**–**c**) normalized second-order guided-wave nonlinearity for specimens loaded at 2, 5, and 10 Hz and (**d**–**f**) corresponding pulse-echo mean amplitude ratios. The guided-wave nonlinearity decreased during relaxation, whereas the pulse-echo amplitude ratio increased as the transient internal stress state was redistributed. Adapted from [27,28].

The second-harmonic amplitude increased across successive fatigue stages, demonstrating progressive growth of acoustic nonlinearity with accumulated loading. This increase was accompanied by a shift in the second harmonic toward a lower frequency. Both accumulated damage and fatigue-induced internal stresses can reduce the effective wave velocity and shift the fundamental and corresponding harmonic components toward lower frequencies. During unloaded relaxation, the second-harmonic amplitude decreased and its frequency shifted partially toward a higher value. These opposing changes established that the nonlinear response contained both a growing component associated with fatigue accumulation and a dissipative component associated with stress relaxation. The differences between the unrelaxed and relaxed nonlinear damage indices are summarized in Figure 13, while Figure 14 and Figure 15 show how the underlying spectral and nonlinear responses changed throughout the 8 h relaxation period.

The magnitude of the dissipated response depended on fatigue stage and loading frequency. After 75,000 cycles, $\tilde{\beta }$ decreased by approximately 12.3%, 19.0%, and 15.2% during the 8 h relaxation period for the 2, 5, and 10 Hz specimens, respectively. After 150,000 cycles, the corresponding reductions were approximately 14.1%, 15.4%, and 17.0%. The largest reductions occurred after 225,000 cycles, reaching approximately 23.1% at 2 Hz, 26.8% at 5 Hz, and 23.4% at 10 Hz. These results demonstrate that the time-dependent contribution to the nonlinear response became more pronounced as fatigue loading accumulated. The maximum reduction of approximately 27% occurred in the 5 Hz specimens after the final fatigue stage [27].

Despite the reduction observed within each relaxation period, the nonlinear response retained after 8 h increased across successive fatigue stages. Relaxation therefore did not return the specimens to their pristine ultrasonic state. Instead, each loading block established a higher persistent level of nonlinearity upon which the next transient relaxation response was superimposed. This distinction shows that the decreasing response within an individual relaxation period should not be interpreted as reversal of fatigue damage. It represents partial dissipation of the transient stress-related contribution, whereas the increasing relaxed-state baseline reflects a persistent response associated with cumulative microstructural degradation.

The response also depended non-monotonically on fatigue loading frequency. The 5 Hz specimens generally exhibited the highest second-order nonlinearity and the greatest reduction during relaxation, indicating both greater accumulated degradation and a larger time-dependent contribution. The 2 Hz specimens showed lower persistent nonlinearity, while the 10 Hz specimens produced an intermediate response. Thus, fatigue severity was governed not simply by loading frequency alone but by the interaction among cyclic loading rate, viscoelastic deformation, stress redistribution, and microstructural damage accumulation.

The pulse-echo measurements showed a consistent time-dependent response. The mean amplitude ratio increased during relaxation, but the duration over which its increment remained at least 0.2% differed among the loading conditions. This period was approximately 1 h for the 2 Hz specimens, 4 h for the 5 Hz specimens, and 2 h for the 10 Hz specimens. The 5 Hz specimens also exhibited the lowest initial mean amplitude ratio, consistent with the most degraded material state. The agreement between the decreasing Lamb wave nonlinearity and increasing pulse-echo amplitude ratio supports the interpretation that material relaxation contributed substantially to the observed temporal response. The sensor-independent pulse-echo results also reduce, but do not eliminate, uncertainty associated with the bonded PZT system.

### 7.4. Implications of Stress Relaxation Assessment

The relaxation experiments demonstrate that ultrasonic indicators of precursor damage depend on the time elapsed between mechanical loading and measurement. An elevated nonlinear response recorded immediately after fatigue loading represents the combined influence of accumulated microstructural degradation and a transient internal stress state. If this time dependence is not considered, changes caused by recoverable stress redistribution may be attributed entirely to persistent damage-related change, producing inconsistent comparisons among experiments or in-service measurements obtained at different post-loading times.

A controlled relaxation period provides a practical means of distinguishing these contributions. The reduction in $\tilde{\beta }$ during relaxation quantifies the dissipative portion of the nonlinear response, while the progressive increase in the relaxed-state value across fatigue stages identifies the persistent component associated predominantly with accumulated degradation. The companion pulse-echo measurements confirm the same temporal behavior without relying on bonded sensors. Together, these observations establish measurement timing as an essential experimental variable and show that precursor damage assessment should compare ultrasonic signals acquired under consistent loading and relaxation conditions. The supporting mechanical and microstructural evidence used to validate the persistent ultrasonic trends is examined in Section 8.

## 8. Experimental Validation of Precursor Damage Indicators

The ultrasonic indicators presented in the preceding sections quantify changes in wave propagation, harmonic generation, spectral modulation, and post-fatigue relaxation. Although these responses are sensitive to the evolving material state, their interpretation requires independent evidence that measurable physical degradation has developed within the laminate. Validation was therefore performed at three complementary levels: residual tensile strength was used to quantify the loss of load-carrying capacity, optical microscopy was used to identify the associated microstructural damage modes, and scanning acoustic microscopy was used to examine internal changes that were not accessible from the specimen surface. Together, these measurements established the mechanical and physical significance of the ultrasonic precursor-damage indicators.

### 8.1. Validation Through Residual Tensile Strength

Residual tensile strength provided a direct measure of the degradation accumulated during fatigue loading. After 300,000 cycles, the CWI specimen remained free of visible macroscopic damage but failed at approximately 790 MPa, compared with approximately 950 MPa for the corresponding pristine reference. This single-specimen comparison represents a strength reduction of approximately 17% and should be interpreted as supporting mechanical evidence rather than a population-level estimate. The result confirms that measurable loss of load-carrying capacity occurred during the regime monitored by CWI [9].

In the separate coordinated Route II study, the three-stage fatigue experiments provided a direct comparison among the 2, 5, and 10 Hz loading conditions. The average tensile strength of the corresponding pristine specimens was approximately 995 MPa. After 225,000 cycles, the remaining strengths decreased to approximately 950 MPa for the 2 Hz specimens, 889 MPa for the 5 Hz specimens, and 939 MPa for the 10 Hz specimens. These values correspond to reductions of approximately 4.5%, 10.7%, and 5.6%, respectively. Thus, the 5 Hz specimens experienced more than twice the strength reduction measured for the 2 Hz specimens and approximately twice that measured for the 10 Hz specimens.

**Figure 16:**
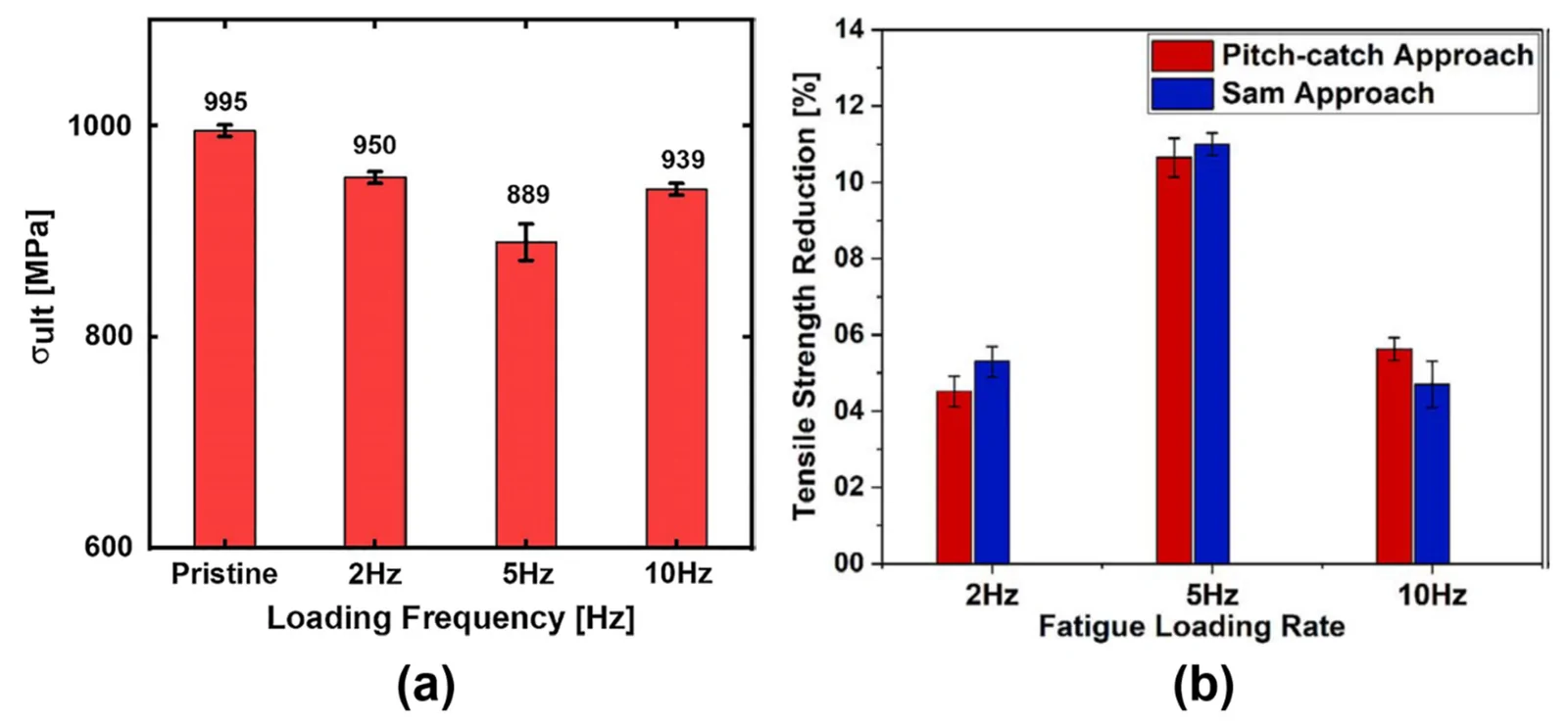
Mechanical validation after 225,000 fatigue cycles: (**a**) remaining tensile strengths of the pristine specimens and specimens loaded at 2, 5, and 10 Hz and (**b**) percentage strength reductions reported for the pitch-catch and pulse-echo specimen subsets. The measurements belong to the coordinated Route II validation dataset and are not treated as independent replications. Within the tested conditions, the 5 Hz group exhibited the greatest loss of load-carrying capacity. Adapted from [27,28].

Comparable loading-frequency trends were observed in the pitch-catch and pulse-echo specimen subsets. For the 5 Hz condition, the reported residual-strength reductions were approximately 11.0% and 11.2%, respectively. These values originated from the coordinated Route II validation dataset reported across the source publications and should not be interpreted as independent replication of the same result. Their agreement supports internal consistency between the specimen subsets, but the small group size does not establish population-level repeatability or statistical equivalence [28]. Figure 16 summarizes the residual-strength comparisons.

The absolute pristine strengths differed between the incremental CWI and three-stage fatigue investigations because they were obtained from separate experimental campaigns and specimen groups. The validation should therefore be interpreted through the strength reduction measured relative to the corresponding pristine specimens rather than through direct comparison of absolute strength between campaigns. Both investigations nevertheless established the same central result: measurable degradation of tensile strength developed during the precursor regime before macroscopic failure or extensive visible delamination.

### 8.2. Optical Microscopy Evidence of Fatigue-Induced Damage

Optical microscopy confirmed that the residual-strength reduction was associated with distributed microstructural damage. In the incremental CWI specimens, the pristine laminate contained only a limited number of manufacturing voids, generally on the order of approximately 5 μm. Following fatigue loading, the density and dimensions of internal discontinuities increased. Matrix cracking, fiber breakage, fiber–matrix separation, and localized interlaminar damage were observed at approximately 160,000 and 300,000 cycles. The matrix cracks reached an average length of approximately 224 μm, although extensive edge delamination and other macroscopic damage remained absent.

Cross-sectional examination after 300,000 cycles revealed pre-delamination sites, fiber separation, fiber–matrix debonding, void development associated with local fiber displacement, and immature interlaminar cracks connecting adjacent matrix cracks. These observations confirmed that multiple damage mechanisms had initiated and interacted within the laminate even though the specimen remained visually intact at the structural scale. The microscopy results therefore support the interpretation of the CWI response as a measure of distributed precursor damage rather than conventional macroscopic defect detection.

**Figure 17:**
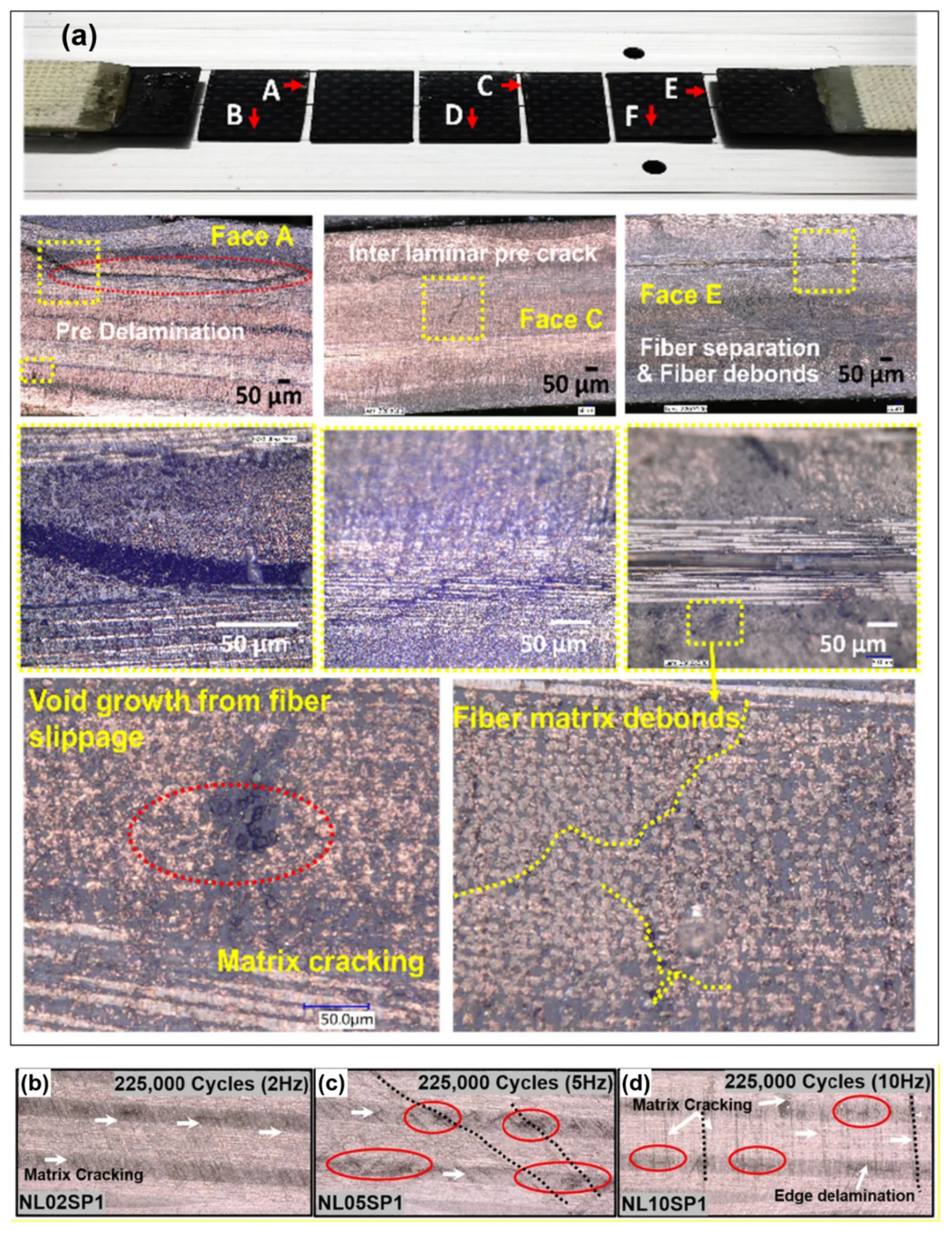
Optical microscopy validation of fatigue-induced precursor damage in CFRP specimens: (**a**) sectioning locations and corresponding micrographs of the decommissioned CWI specimen after 300,000 cycles, showing pre-delamination, immature interlaminar cracking, fiber separation, fiber–matrix debonding, void growth, and matrix cracking; and (**b**–**d**) cross-sectional observations after 225,000 cycles at 2, 5, and 10 Hz, respectively. The 5 Hz specimen exhibited the greatest concentration of matrix cracking and interfacial damage. Adapted from [9,30].

The specimens subjected to the three-stage fatigue protocol exhibited the same general damage modes after 225,000 cycles. Matrix microcracking, isolated fiber fracture, fiber–matrix separation, and localized edge delamination were observed for the 2, 5, and 10 Hz conditions. However, their concentrations differed substantially. The 5 Hz specimens contained the greatest density of matrix cracking and interfacial damage, whereas the 2 Hz specimens showed the least extensive degradation. The 10 Hz specimens exhibited an intermediate damage state. This qualitative ranking agreed with the residual-strength measurements and with the loading-rate dependence identified by the nonlinear ultrasonic parameters. Figure 17 compiles the microscopy evidence obtained from the 300,000-cycle CWI specimen and the specimens subjected to 225,000 cycles at 2, 5, and 10 Hz.

The microscopy findings also clarify the physical interpretation of the nonlinear parameters. The increase in $\tilde{\beta }$ is consistent with the greater prevalence of interfacial separation, crack-edge interaction, and local contact behavior, while the reduction in $\tilde{\gamma }$ is consistent with disruption of the matrix-dominated nonlinear elastic response. These associations do not imply that either parameter uniquely identifies an individual damage mechanism. Rather, the opposing trends reflect the combined influence of the damage populations observed within the heterogeneous laminate.

### 8.3. Scanning Acoustic Microscopy and Material-State Mapping

Optical microscopy requires specimen sectioning and provides information at the examined cross sections. Scanning acoustic microscopy supplied complementary evidence by mapping internal ultrasonic properties over a broader region without mechanically sectioning the specimen. In the incremental CWI investigation, high-resolution SAM images acquired in the pristine state and after approximately 160,000 and 300,000 cycles showed progressive development of matrix cracking and interfacial debonding. The locations and stages at which these features became visible were consistent with changes identified from the CWI response. SAM examination of the decommissioned specimen also revealed pre-delamination sites and immature interlaminar damage beneath regions that showed no macroscopic surface defect.

A separate SAM investigation examined the through-thickness velocity distributions obtained from approximately 8750 spatial locations after 75,000, 150,000, and 225,000 cycles. For the 5 Hz specimens, the velocity distribution after 75,000 cycles remained comparatively narrow, whereas its spread increased substantially after 150,000 and 225,000 cycles. The increasing spatial dispersion indicated that fatigue loading generated a progressively more heterogeneous internal material state rather than a uniform reduction in stiffness.

The velocity distributions also responded to loading frequency. Specimens tested at 15 Hz showed broader distributions and greater material-state disorganization than those tested at 5 Hz within that experimental campaign. Because the 5 and 15 Hz entropy study used a different specimen set and loading-rate comparison from the 2, 5, and 10 Hz investigations, its results are not used to establish a common ranking across all experiments. Instead, they provide separate supporting evidence that the spatial distribution of through-thickness wave velocity becomes increasingly heterogeneous with accumulated fatigue loading and depends on the applied loading rate [26].

**Figure 18:**
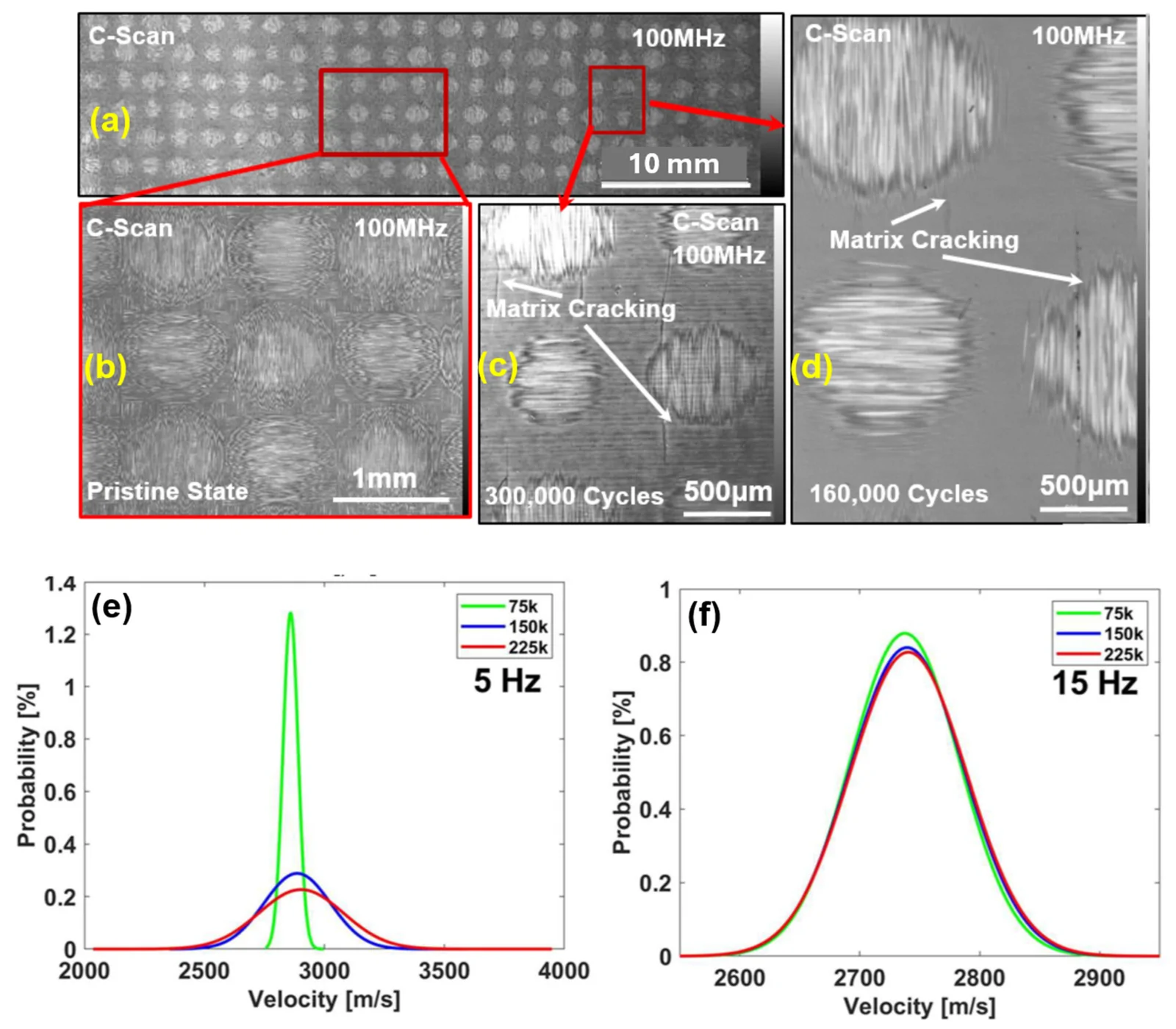
Scanning acoustic microscopy evidence of internal material-state changes: (**a**–**d**) representative SAM images in the pristine condition and after ~ 160,000 and 300,000 fatigue cycles and (**e**,**f**) probability distributions of through-thickness ultrasonic velocity measured after 75,000, 150,000, and 225,000 cycles at 5 and 15 Hz, respectively. The development of matrix cracking and interfacial damage was accompanied by increasing spatial dispersion of ultrasonic velocity. Adapted from Patra and Banerjee [9,26].

The probability distributions of the velocity maps were further expressed through a damage-entropy parameter. Both the spread of the velocity distribution and the corresponding entropy increased with accumulated fatigue cycles, indicating progressive internal disorganization. During the subsequent unloaded periods, both quantities decreased as internal stresses redistributed and the measured velocities partially recovered. This response independently supports the distinction developed in Section 7 between the persistent material degradation accumulated across fatigue stages and the time-dependent component dissipated during post-fatigue relaxation. Figure 18 combines the SAM images from the incremental CWI investigation with the through-thickness velocity distributions obtained from the separate fatigue-relaxation investigation.

### 8.4. Consistency Among Ultrasonic and Independent Damage Measures

The mechanical and microstructural observations provide convergent validation of the ultrasonic precursor-damage indicators. Within the three-stage 2, 5, and 10 Hz experiments, the guided-wave and high-frequency measurements consistently identified the 5 Hz specimens as the most degraded condition. These specimens exhibited the strongest guided-wave nonlinear response, the highest high-frequency $\tilde{\beta }$, the lowest high-frequency $\tilde{\gamma }$, the lowest cumulative sideband peak count, the greatest concentration of observed microstructural damage, and the lowest residual tensile strength. The incremental CWI campaign similarly showed measurable ultrasonic changes before macroscopic damage, followed by microscopic evidence of matrix cracking, interfacial separation, and pre-delamination.

These comparisons validate the ultrasonic trends without implying a unique relationship between any individual indicator and a specific damage mechanism. Residual strength quantifies mechanical consequence, microscopy identifies local damage modes, and SAM maps internal heterogeneity. Their combined interpretation provides stronger validation than any individual comparison.

## 9. Comparative Assessment of Ultrasonic Techniques

The preceding studies demonstrate that precursor fatigue damage cannot be represented adequately by a single ultrasonic quantity. Coda-wave interferometry, guided-wave nonlinear analysis, high-frequency pulse-echo measurements, and SAM-based material-state mapping respond to different consequences of the same developing damage state. Their relative value therefore depends on the required inspection scale, measurement frequency, structural accessibility, and whether the objective is detection, localization, material characterization, or separation of transient stress-related and persistent damage-related responses.

### 9.1. Comparison of Sensing Mechanisms

The techniques investigated differ in sensing mechanism, inspection scale, and deployment requirements. Table 2 compares the sensing mechanisms, inspection scales, reported quantitative evidence, limitations, and most appropriate roles of the five assessment approaches.

### 9.2. Sensitivity Across Spatial and Temporal Scales

The techniques differ principally in how they sample the material. CWI and guided-wave nonlinear ultrasonics integrate changes over the in-plane propagation path between bonded sensors. CWI increases sensitivity through multiple scattering within the coda, whereas guided-wave nonlinear analysis uses spectral energy transferred to higher harmonics. High-frequency P-waves interrogate a much smaller through-thickness region, but spatial scanning extends the measurement over thousands of locations and produces a map of local material-state variability.

The methods should not be arranged as a universal sensitivity hierarchy because they sample different spatial domains and physical responses. CWI provides frequent measurements of distributed propagation changes, guided wave nonlinearity assesses harmonic interactions over an extended path, and scanned pulse-echo measurements resolve localized through-thickness variations. Their value depends on whether the objective is early indication, structural coverage, local confirmation, or material state mapping.

Temporal scale is equally important. CWI measurements collected during incremental loading characterize progressive changes between fatigue states, whereas the Route II measurements also captured the response during unloaded relaxation. Consequently, differences among measurements acquired at unequal post-loading times reflect stress redistribution as well as damage accumulation. Relaxation is therefore not an additional competing technique but a temporal dimension that must be incorporated into the interpretation of every ultrasonic indicator sensitive to internal stress.

### 9.3. Measurement Uncertainty and Deployment Considerations

Bonded guided-wave sensors support continuous structural health monitoring but introduce uncertainty associated with sensor response and adhesive integrity. CWI is particularly sensitive to small waveform perturbations, meaning that temperature, boundary conditions, applied load, and bond condition can produce changes comparable to those generated by precursor damage. Guided-wave harmonic analysis introduces an additional requirement to control system nonlinearity and maintain consistent excitation amplitude, frequency, and mode content. Baseline monitoring of pristine specimens and periodic sensor diagnostics are therefore necessary for both approaches.

High-frequency pulse-echo measurements avoid uncertainty from permanently bonded sensors, but they introduce other sources of variability. Transducer alignment, water-path length, focal position, coupling conditions, and spatial registration must remain consistent among scans. Harmonic amplitudes and sideband counts are also influenced by baseline noise, spectral window selection, and threshold definition. The wavelet-based normalization and statistical fitting used in the high-frequency studies reduced these effects, but such processing increases computational and procedural complexity.

The techniques also differ in readiness for structural deployment. Bonded CWI and guided-wave nonlinear measurements can be implemented using compact permanently installed sensor networks. High-frequency pulse-echo and SAM provide more detailed localized information but presently require access to the inspection surface, controlled coupling, and scanning equipment. Thus, the technique with the greatest local sensitivity is not necessarily the most practical for continuous monitoring, while the technique with the largest coverage may not provide sufficient spatial or mechanistic resolution for a complete diagnosis. These distinctions provide the basis for the monitoring and confirmation strategy proposed in Section 10.

## 10. Toward an Integrated Strategy for Precursor Damage Assessment

The findings support a staged assessment strategy rather than simultaneous use of every technique. Bonded guided wave sensors provide routine structural monitoring, while high-frequency pulse-echo and SAM provide targeted confirmation when a persistent indication requires further examination. Mechanical testing and microscopy remain laboratory validation methods rather than components of operational monitoring.

### 10.1. Continuous Monitoring Using Guided Waves

CWI and guided-wave nonlinear ultrasonics can be implemented using the same bonded actuator–receiver network, although analysis-specific excitation and acquisition settings may be required. They therefore represent complementary analyses supported by a common sensor network rather than separate monitoring systems [15,39]. CWI uses the multiply scattered coda to identify changes in effective wave velocity across a distributed region, whereas guided-wave nonlinear analysis uses the harmonic content of the direct propagating wavefield to identify nonlinear changes associated with microstructural degradation.

The two analyses answer different questions. A progressive change in PDI indicates that the distributed material state has changed relative to the preceding measurement. A corresponding change in guided-wave harmonic parameters provides additional evidence that the response includes nonlinear material behavior. Agreement between these indicators increases confidence in the assessment, but strict agreement should not be imposed as a universal decision rule because their physical sensitivities differ. A CWI indication without a comparable harmonic change may still represent a developing material-state perturbation and should prompt examination of environmental conditions, sensor integrity, and subsequent measurement trends.

The combined use of CWI and guided-wave nonlinear analysis represents the most practical outcome of the synthesized studies for in-service monitoring [40,41]. Both indicators can be obtained using the same bonded sensor network, provided that the excitation conditions, sensor condition, temperature, applied load, boundary conditions, and measurement timing are documented consistently. The approach extends conventional guided-wave monitoring from detection of established defects toward surveillance of gradual material-state changes during the precursor regime [9,27,30,42].

### 10.2. Targeted Confirmation Using High-Frequency Ultrasonics

High-frequency pulse-echo and SAM should be considered targeted inspection methods rather than mandatory components of continuous monitoring. When guided-wave measurements indicate a persistent change, high-frequency scanning can be applied to structurally critical regions or to the region covered by the affected actuator–receiver path. Through-thickness harmonic parameters, sideband peak counts, reflection amplitudes, and velocity distributions can then determine whether localized internal changes are present without relying on the bonded sensor network.

This separation of roles addresses the different capabilities of the two configurations. Guided waves provide efficient coverage of a relatively large structural region but integrate the effects of multiple damage mechanisms along their propagation paths. High-frequency pulse-echo provides greater spatial resolution and sensor-independent material interrogation but requires access to the inspection surface and controlled coupling. It is therefore more appropriate for periodic inspection, laboratory characterization, or follow-up examination after a guided-wave indication.

**Figure 19:**
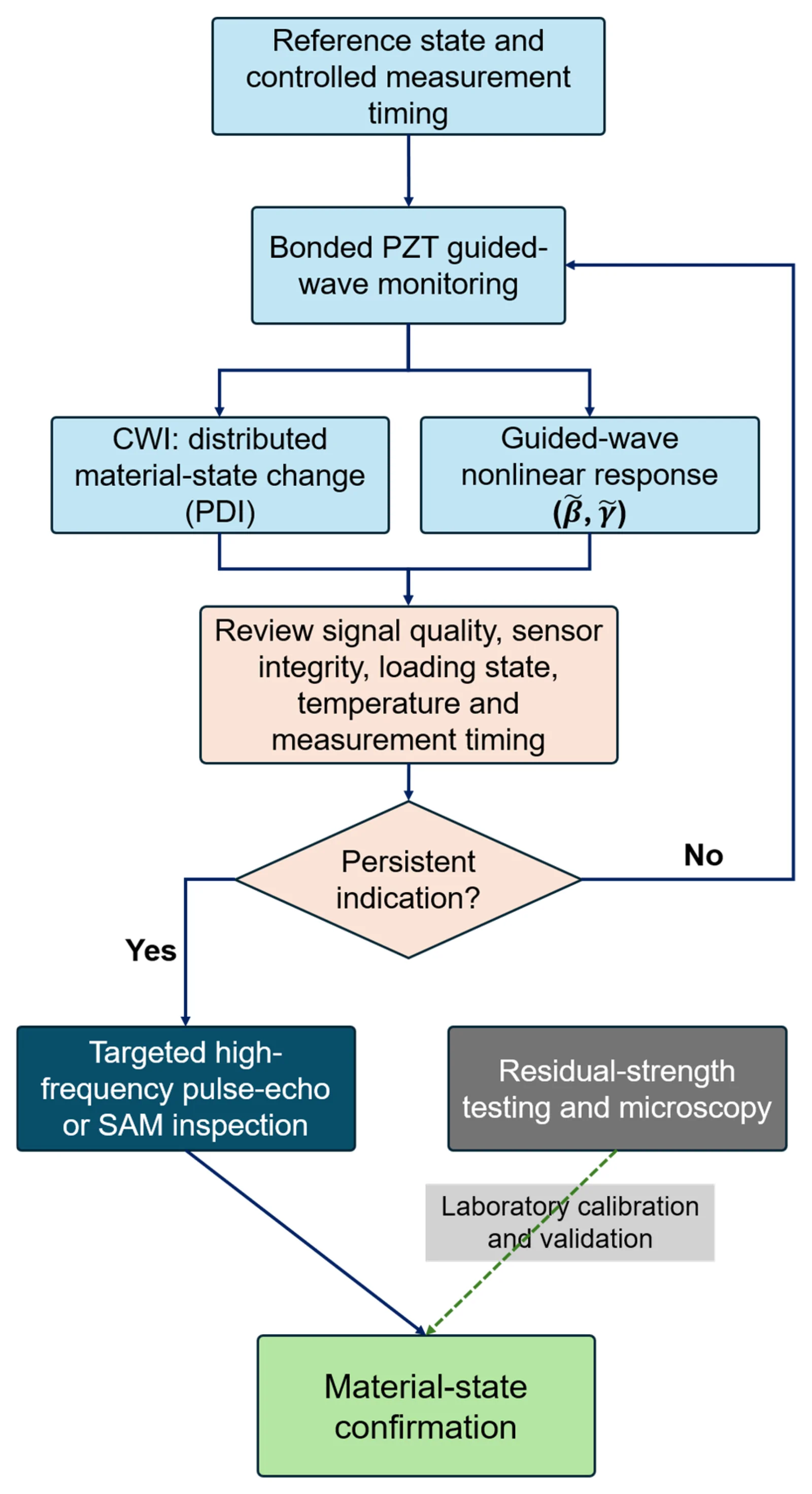
Proposed staged strategy for precursor damage assessment in CFRP composites. A bonded PZT network supports structural-scale monitoring through CWI and guided-wave nonlinear analysis. Indications that persist after evaluation of signal quality, sensor integrity, temperature, loading state, and measurement timing motivate targeted high-frequency pulse-echo or SAM inspection. Residual-strength testing and microscopy provide laboratory calibration and validation of the ultrasonic indicators but are not components of routine in-service monitoring.

The high-frequency measurements also provide an important cross-check against sensor-related uncertainty. If bonded guided-wave indicators and independently acquired high-frequency pulse-echo measurements show comparable material-state changes, confidence increases that the response originates within the composite rather than from the PZT sensors or adhesive layers. Spatial velocity and entropy distributions obtained through SAM further indicate whether the internal material state has become increasingly heterogeneous with fatigue loading [26,28,29]. These monitoring, confirmation, and validation roles are integrated into the staged assessment strategy presented in Figure 19.

### 10.3. Measurement Timing and Cross-Validation

Measurement timing forms part of the assessment protocol because signals acquired immediately after unloading contain contributions from accumulated degradation and transient internal stress. Comparisons should therefore be made at defined post-loading times or under comparable operational states. The 8 h interval used in the reported experiments is not proposed as a universal requirement; the appropriate interval must be established for the material system, loading history, and operating environment.

Residual-strength testing, microscopy, and high-resolution SAM serve as laboratory calibration methods rather than components of routine monitoring. Their role is to relate the ultrasonic indicators to internal damage and loss of structural capacity. The proposed strategy therefore uses guided waves for initial monitoring, controlled timing to improve interpretation, and localized pulse-echo or SAM measurements for follow-up confirmation. This sequence remains conceptual and requires validation on representative structural components before operational thresholds can be defined.

## 11. Current Challenges and Future Research Directions

The six studies synthesized in this paper demonstrate that coda wave interferometry, nonlinear guided waves, and high-frequency pulse-echo measurements can detect progressive material state changes before conventional ultrasonic features indicate substantial damage. However, several challenges must be addressed before these indicators can support routine structural assessment.

### 11.1. Generalizability Across Materials and Loading Conditions

The reported relationships were established for specific CFRP material systems, laminate configurations, specimen geometries, and fatigue conditions. Because ultrasonic propagation and nonlinear response are influenced by anisotropy, stacking sequence, thickness, resin properties, and defect morphology, the observed parameter trends should not be treated as universal calibration relationships [14,15]. Fatigue damage evolution also depends on stress ratio, loading frequency, and load sequence, which continue to limit the transferability of composite fatigue models [8]. Future studies should therefore evaluate larger specimen populations and additional material systems to determine which ultrasonic trends are physically generalizable and which require application-specific calibration.

### 11.2. Separation of Damage from Environmental and Operational Effects

Temperature, applied stress, moisture, and boundary conditions can modify ultrasonic velocity, amplitude, phase, modal content, and transducer response without producing persistent damage-related change [43,44]. These effects are particularly important for coda wave and nonlinear measurements because the changes associated with precursor damage may be small. Ren et al. [16] demonstrated that the temperature response of embedded PZT measurements depends on frequency, wave mode, and laminate thickness. Future work should establish environmental compensation procedures and uncertainty bounds before changes in PDI, harmonic amplitudes, or sideband features are attributed to damage. Measurements collected after controlled relaxation provide one practical approach, although continuous monitoring will require separation of reversible operational effects from persistent damage-related material response.

### 11.3. Measurement Repeatability and Sensing System Contributions

The response of a bonded PZT measurement system depends on transducer capacitance, adhesive thickness and integrity, sensor position, cable and connector condition, excitation amplitude and frequency, receiver bandwidth, and instrumentation nonlinearity. In Route II, the adhesive layer was controlled to approximately 150 μm, sensor capacitance was monitored, and the same 512-point moving-average filter was applied consistently to the recorded waveforms [28,29]. The pristine control exhibited less than approximately 0.1% variation during the 8 h observation period. These controls support short-term laboratory stability and indicate that the largest reported relaxation-related changes, which reached 12.3–26.8%, exceeded the measured pristine drift. They do not, however, quantify specimen-to-specimen, installation-to-installation, or long-term sensor-aging variability.

Field implementation will require baseline impedance or capacitance measurements, reference propagation paths, cable and connector diagnostics, repeated installation studies, control of excitation-system nonlinearity, temperature compensation, and objective criteria for identifying sensor debonding or failure. Changes in sensor coupling can resemble changes in the host structure; therefore, sensor-health information should be evaluated before an ultrasonic indicator is interpreted as material degradation.

### 11.4. Localization and Damage Mechanism Attribution

The investigated parameters indicate that the material state has changed, but they do not uniquely determine the location or physical mechanism of damage. Similar changes may arise from matrix cracking, interface degradation, frictional contact, isolated fiber fracture, or delamination. Time–frequency analysis of diffuse ultrasonic signals has shown that damage sensitivity can be concentrated within repeatable regions of the measured response, providing a possible route toward improved discrimination [45]. Sensor networks and probabilistic imaging have also demonstrated guided wave localization in a realistic CFRP fuselage panel [46,47,48]. Future studies should combine ultrasonic indicators with spatially resolved methods such as scanning acoustic microscopy or X-ray computed tomography before assigning individual nonlinear features to specific damage mechanisms.

### 11.5. Relationship with Residual Structural Capacity

Detection of precursor damage does not by itself establish its structural significance. Four assessment levels must be distinguished. Detection identifies a change from the baseline material state. Localization determines where the change occurred. Quantification relates the measured indicator to damage severity. Prognosis estimates future damage development, residual strength, remaining fatigue life, or probability of failure. The present evidence is strongest for detection and material-state characterization. The scanned measurements also provide spatial maps of ultrasonic variation, but the available results do not establish a general relationship between indicator magnitude and damage severity.

An engineering assessment requires a quantitative relationship between the measured ultrasonic response and residual strength, remaining fatigue life, or probability of failure [49]. The residual strength and microscopy results in this synthesis provide initial evidence that the ultrasonic changes have mechanical and microstructural significance, but the dataset does not support a broadly applicable prognostic model. Current CFRP fatigue predictions remain sensitive to material system, loading history, damage representation, and dataset size [8]. Future studies should determine whether combining PDI, nonlinear parameters, sideband features, and relaxation behavior improves residual-capacity and fatigue-life estimation compared with any individual indicator.

### 11.6. Complementary Sensing and Data Integration

Other sensing methods can provide information that is not available from ultrasonic measurements alone. Acoustic emission records transient elastic waves generated during active damage development and can support event localization when a sensor array is used. Its effectiveness depends on the occurrence of active damage events and on the ability to separate damage signals from operational noise [50]. Broadband dielectric spectroscopy measures changes associated with moisture uptake, polymer mobility, and matrix or interface condition. It may therefore help distinguish environmental exposure from mechanically induced changes in the ultrasonic response [51]. Electrical-resistance self-sensing has also been combined with convolutional neural networks to identify damage location and severity in CFRP structures [52]. These methods respond to different physical processes and may complement ultrasonic assessment.

Data-driven analysis could combine CWI velocity changes, nonlinear harmonic parameters, sideband counts, relaxation behavior, acoustic-emission features, electrical resistance, dielectric response, and operational variables. The present specimen population is not sufficient to train or independently validate such a model. Future studies will require larger datasets, independent validation specimens, uncertainty reporting, and testing on representative structural components. Field studies using dynamic-response measurements and regression-based fatigue-life models demonstrate how monitoring data can support structural assessment at larger scales [53,54]. These studies concern bridge structures rather than CFRP ultrasonic measurements and are included to illustrate possible routes toward field validation and prognosis.

### 11.7. Translation to Structural Monitoring

Implementation of structural components will require sensor network optimization, automated signal quality assessment, environmental compensation, and defined detection thresholds. Monitoring reliability must also account for sensor degradation or failure because changes in the sensing network can reduce the information available for damage assessment. Wu et al. [55] demonstrated a formal approach for quantifying this loss of SHM reliability in ultrasonic guided wave systems. Validation on a realistic CFRP fuselage panel further illustrates the importance of testing monitoring methods on complex structural geometries rather than coupons alone [46,56].

The immediate research priority is to determine the robustness, transferability, and prognostic value of the indicators examined in this synthesis. Larger independent datasets and structural-scale validation are needed before these indicators can be evaluated for use in residual-capacity assessment or structural decision-making.

## 12. Conclusions

This synthesis shows that fatigue-induced material-state changes in woven CFRP can produce measurable ultrasonic responses before visible delamination or major alteration of the first-arriving guided-wave packet. Incremental CWI detected distributed propagation changes, nonlinear guided waves measured harmonic response over the actuator–receiver path, and high-frequency pulse-echo and SAM measurements provided localized through-thickness characterization. The nonlinear guided-wave response decreased by as much as 26.8% during the 8 h unloaded period, demonstrating that post-loading measurement time must be controlled when transient stress-related and persistent damage-related responses are compared. Within the specific Route II material system and loading protocol, the 5 Hz specimens exhibited the greatest degradation among the 2, 5, and 10 Hz conditions; this ranking is not proposed as a general frequency–damage relationship for CFRP composites. Residual-strength testing, microscopy, and SAM provided convergent supporting evidence, but the overlapping datasets and small specimen groups do not constitute independent statistical replication. The proposed framework therefore demonstrates laboratory-scale sensitivity to precursor material-state changes. It does not yet provide reliable prediction of residual strength, remaining fatigue life, or probability of failure. Field implementation will require larger independent specimen populations, uncertainty and threshold estimation, repeated sensor installations, environmental compensation, validation under variable loading histories, and demonstration on representative structural components.

## Figures and Tables

**Table 1:** Source studies, specimen allocation, experimental conditions, and dataset relationships within the present synthesis.

Source Study	Route	Fatigue Condition	Method	Specimen Allocation	Dataset Relationship
Patra and Banerjee [9]	I	10 Hz; measurements to 300,000 cycles	Guided CWI	Five CWI specimens; one separate specimen used for residual-strength comparison	Separate incremental CWI campaign
Ahmed et al. [26]	II	5 and 15 Hz	SAM velocity and entropy	Three SAM specimens per frequency	Separate 5/15 Hz experiment; must not be pooled quantitatively with the 2/5/10 Hz campaign
Patra et al. [27]	II	2, 5, and 10 Hz	Nonlinear guided waves	Nine pitch-catch specimens; *n* = 3 per frequency	Pitch-catch subset of the 22-specimen program
Patra et al. [28]	II	2, 5, and 10 Hz	Guided waves, pulse-echo/SAM, and residual strength	Nine pitch-catch + nine pulse-echo/SAM + three pristine tensile specimens; total reported *n* = 21	The fatigue-life specimen was not included in this publication
Ahmed et al. [29]	II	2, 5, and 10 Hz; three 75,000-cycle stages	CWI and nonlinear ultrasonics	Nine pitch-catch + nine pulse-echo/SAM + three pristine tensile + one fatigue-life specimen; total prepared *n* = 22	Coordinated Route II program
Ahmed et al. [30]	II	2, 5, and 10 Hz	High-frequency pulse-echo and SAM	Nine pulse-echo/SAM + three pristine tensile + one fatigue-life specimen; reported subset *n* = 13	High-frequency subset of the same 22-specimen program

**Table 2:** Comparative characteristics of the ultrasonic approaches used for precursor damage assessment in CFRP composites.

Approach	Principal Measurement	Inspection Scale	Primary Strength	Principal Limitation	Most Appropriate Role
CWI	Relative velocity variation and PDI	Distributed guided-wave region	Five specimens; 61 monitored material states; measurable activity at approximately 120,000–160,000 cycles	Limited localization and mechanism specificity	Continuous detection and trend monitoring
Guided-wave nonlinear ultrasonics	$\tilde{\beta }$, $\tilde{\gamma }$, and higher-harmonic frequency shifts	Extended actuator–receiver path	Three specimens per frequency; time-dependent reductions reached 12.3–26.8% after 225,000 cycles	Sensitive to instrumentation, bonding, mode selection, and excitation conditions	In situ confirmation of nonlinear degradation
High-frequency pulse-echo	Harmonic amplitudes, $\tilde{\beta }$, $\tilde{\gamma }$, and sideband peak count	Localized through-thickness region	Three specimens per frequency; 25 MHz interrogation over 9375 spatial locations per specimen	Attenuation, limited coverage, and immersion requirements	Local characterization and periodic inspection
SAM material-state mapping	Velocity distributions, reflection ratios, and damage entropy	Scanned two-dimensional region	Three specimens per frequency; approximately 8750 spatial locations and 33 time states per relaxation period	Time-intensive scanning under controlled conditions	Detailed mapping and independent validation
Relaxation-based assessment	Change between unrelaxed and relaxed ultrasonic states	Temporal measurement dimension	Measurements every 15 min for 8 h; maximum measured reduction of 26.8%	Requires controlled timing and repeated measurements	Standardization and interpretation of damage indicators

## Data Availability

No new experimental data were generated specifically for this synthesis. The data supporting the findings discussed in this article are reported in the cited source publications. Additional information may be obtained from the corresponding author upon reasonable request.
